# Seventh meeting of the Global Alliance to Eliminate Lymphatic Filariasis: reaching the vision by scaling up, scaling down, and reaching out

**DOI:** 10.1186/1756-3305-7-46

**Published:** 2014-01-23

**Authors:** Molly Brady

**Affiliations:** 1 RTI International, 701 13 St NW, Suite 750, Washington DC 20005, USA; 2 Centre for Neglected Tropical Diseases, Liverpool School of Tropical Medicine, Pembroke Place, Liverpool L3 5QA, UK

**Keywords:** Lymphatic filariasis, Neglected tropical diseases, Review

## Abstract

This report summarizes the 7^th^ meeting of the Global Alliance to Eliminate Lymphatic Filariasis (GAELF), Washington DC, November 18–19, 2012. The theme, “A Future Free of Lymphatic Filariasis: Reaching the Vision by Scaling Up, Scaling Down and Reaching Out”, emphasized new strategies and partnerships necessary to reach the 2020 goal of elimination of lymphatic filariasis (LF) as a public-health problem.

## Executive summary

The 7^th^ Meeting of the Global Alliance to Eliminate Lymphatic Filariasis (GAELF) was held 18–19 November, 2012 in Washington, DC. The theme of the meeting, “A Future Free of Lymphatic Filariasis: Reaching the Vision by Scaling Up, Scaling Down and Reaching Out”, emphasized new strategies and partnerships necessary to reach the 2020 goal of elimination of lymphatic filariasis (LF) as a public-health problem. The meeting was held at the World Bank, back to back with a Uniting to Combat Neglected Tropical Diseases (NTDs) forum, in order to capitalize on common themes, participants, and support (http://www.unitingtocombatntds.org).

## Current status

The World Health Organization’s overall goal is universal health coverage with essential public health interventions, including access to preventive chemotherapy (PC) for NTDs. The goal of integrated PC is to eliminate LF, onchocerciasis, blinding trachoma and, in some cases, schistosomiasis as public-health problems and eliminate childhood morbidity related to soil-transmitted helminthiases (STH). Given that 1.4 billion people require mass drug administration (MDA), a type of PC, for LF, the LF programme’s community-based MDA is often used as the platform for integrating PC diseases. Successful scale up for LF MDA can also cover the majority of the 1.9 billion people needing PC for at least one NTD.

The current status of the Global Programme to Eliminate Lymphatic Filariasis (GPELF) is that 73 countries are endemic, with 1.39 million people at risk, 120 million people infected, and 40 million people affected by LF-related morbidity. The goal of global elimination has two parts: i) to stop the spread of infection and interrupt transmission through MDA and ii) to reduce human suffering through managing morbidity and preventing disability (MMDP). The first part of the goal is realized through the sequential activities of mapping, MDA, post-MDA surveillance and verification. In 2011, 53 countries implemented MDA, with nearly 539 million people treated, and 12 countries under post-MDA surveillance. The second part is achieved through analyzing the MMDP situation in country, developing a plan, and providing access to a minimum package of MMDP care. In contrast to the success at scaling up MDA, only 27 countries have reported MMDP activities.

Progress towards milestones in the GPELF Strategic Plan 2010–2020 has been impressive. Revised M&E guidance, including information on implementing Transmission Assessment Surveys (TASs) and verifying absence of transmission, has been published. A provisional strategy for interrupting transmission in loiasis co-endemic areas, where MDA with ivermectin cannot be implemented due to concerns of serious adverse reactions, will be presented to the Strategic and Technical Advisory Group on NTDs for endorsement in April 2013. Revised MMDP programmatic guidance, including metrics for reporting on MMDP activities to WHO, will be finalized in 2013.

## Country case studies

A series of country case studies highlighted common themes necessary to achieve success and overcome challenges. Success in scaling up was bolstered by cultivating dedicated programme managers, maintaining government commitment at all levels, and building on existing delivery platforms. Presentations on success in scaling down showcased the importance of government support lasting beyond MDA, as well as providing ongoing technical assistance for post-MDA surveillance and dossier development. Countries facing significant challenges were often those in post-conflict situations, with poor infrastructure and weak health care systems. Overcoming these challenges takes innovative thinking about delivery strategies, the creation of novel ways to motivate volunteers, and external financial and technical support. Finally, case studies on accelerating progress showed the importance of improving coverage in areas with ongoing MDA before scaling up, and the critical need to build the capacity of LF programme managers to plan, budget and monitor programmes, especially in large, decentralized countries.

Participants learned about pioneering efforts to manage morbidity and prevent disability, including Togo’s national lymphoedema programme, a community-based lymphoedema project in a highly-endemic area of India, and the African LF Morbidity Project. Providing care for LF-related disease was shown to increase acceptability and compliance with MDA. The group welcomed the results of a clinical trial which showed that doxycycline reversed or halted progression to later stages of lymphoedema more effectively than amoxicillin or placebo, in patients with and without active LF infection. The importance of linking MMDP with other diseases, reporting on MMDP activities to WHO, and collecting data on surgical complications and recurrence was discussed.

A session on linking LF programmes with other disease programmes explored the rationale and opportunities for coordinating with onchocerciasis, STH, trachoma, and malaria activities. Opportunities for synergy with onchocerciasis and trachoma activities include integrated mapping, joint training, combined treatment registers, simultaneous MDA where feasible, and joint M&E. STH activities greatly benefit from the LF programme; in 2010, WHO reported that 57% of the school-aged children treated for STH were given these medicines through national LF programmes. However, like MMDP activities in LF programmes, interventions related to water, sanitation and hygiene often lag behind PC interventions in STH programmes. Both programmes need to bolster these supportive activities if the 2020 goal to eliminate LF is to be achieved. LF and malaria activities also complement each other: LF MDA helps lower anemia, provides community networks for bednet monitoring, and influences adults to use bednets to avoid LF-related disease, while bednets help reduce LF transmission. An animated discussion followed the presentation of data from ongoing entomological monitoring in Nigeria which found no infective mosquitoes after bednet distribution in areas with LF MDA.

## Moving forward

Various speakers highlighted challenges to achieving the 2020 goal, including: i) building the capacity of LF programme managers and NTD teams to plan, implement and monitor activities; ii) scaling up to 100% geographical coverage for MDA while coordinating with other PC diseases and vector control activities; iii) planning for TAS, including ensuring the availability and affordability of diagnostic tests; iv) developing effective post-MDA surveillance tools and methods; and v) scaling up MMDP activities to provide access to all patients.

To respond to these challenges, policy, advocacy, implementation and operational research should be approached using a network of partners, including Ministries of Health, WHO, GAELF, GPELF, academia, and non-governmental development organizations. Developing strategic partnerships based on understanding the motivations of each partner was seen as a key to success to collaboration with other disease programmes, private business, and media organizations. Presenters also stressed the need to build capacity of dynamic programme managers, increase support for MMDP, use integration as an opportunity to scale up, and continue to galvanize government commitment to LF programmes.

The continuing importance of GAELF as one in a mix of NTD alliances was evident, with the GAELF 7 meeting providing an opportunity to celebrate successes, reflect on ongoing challenges, and share information among circa 250 representatives from country programmes, donors, and the research community. The future of GAELF as a forum for discussion and advocacy will be improved by increasing country participation and engagement in the daily work of the GAELF. The group was challenged to continue GAELF’s evolution – already a great example of a ‘mass uprising of compassion’ through its treatment of millions of at-risk people - by maintaining focus on interrupting LF transmission while expanding its peripheral vision to include those with LF-related disease.

## Opening session – are we on track for 2020?

### Welcome by the chair of the GAELF

Dr Patrick Lammie (Centers for Disease Control and Prevention (CDC), USA) welcomed participants to the GAELF meeting, following on from the successful Uniting to Combat NTD forum (Additional file 1). He thanked Dr Don Bundy and his team at the World Bank for hosting the meeting, the Bill & Melinda Gates Foundation (BMGF) team for arranging the meeting venue logistics, and GlaxoSmithKline (GSK) and Merck & Co., Inc. for their drug donations and consistent support of GAELF. He also gave a special welcome to the newest GAELF partner – Eisai Inc. – which will donate diethylcarbamazine (DEC) starting in 2013.

He asked the group to remember two colleagues, Dr Vely Jean Francois, the programme manager for LF and malaria in Haiti and Dr Likezo Mubila, the WHO Africa Region focal point for NTDs, who were at the last GAELF meeting in South Korea, but have since passed away. Dr. Jean Francois’ commitment to improving health was a source of inspiration. During a period of civil strife, he was kidnapped and held for ransom, but upon his release chose to stay in Haiti and continue his public health work. Sadly, he died before seeing the LF programme reach national coverage in 2012. Dr Likezo Mubila, was ‘our conscience’, always reminding the group of the countries and communities left behind. Dr Lammie expressed hope that when GAELF members debate ways to reach our targets in all communities, we hear her voice reminding us of that obligation.

He closed by noting while we can celebrate successes, we need to be mindful about finding new strategies and partnerships to reach the ambitious 2020 objective of elimination of LF.

### Welcome by the Minister of Health, Tanzania

Dr Hussein Mwinyi (Honorable Minister of Health, Tanzania) remarked that it was good to be back among old friends, having been involved in the early days of GAELF. Dr Mwinyi was first struck by the suffering of elephantiasis patients when he was a member of parliament from a highly-endemic district. That experience motivated him to start the Tanzanian LF programme as a way to signal hope for the neglected populations in endemic communities.

He expressed delight at the progress GAELF has made in a relatively short time, including the number of people treated and the number of countries with active programmes. The clear goal of elimination – with the countries at the centre – has resulted in tremendous achievements, although it is essential to upscale faster, especially in parts of sub-Saharan Africa and Indonesia, and monitor progress closely.

He noted many of the outstanding needs of the GAELF, such as improved tools for ongoing post-MDA surveillance, stronger morbidity programmes, strategies for MDA in urban areas, and operational research guided by programme needs. He highlighted how the Tanzanian President established a fund to support LF patients, but there is still a major backlog of hydrocele surgeries needed. He challenged the group to report on progress of morbidity burden assessment and activities at the next GAELF meeting.

Dr Mwinyi emphasized that partnership and coordination are key components of the effectiveness of GAELF, encouraging the group to re-examine what the GAELF can learn and share with other NTD alliances and to use GAELF’s momentum to meet NTD goals.

## Preventive chemotherapy and WHO’s strategic plan for NTDs

Dr Dirk Engels (WHO Department of Control of NTDs, Geneva) discussed how WHO is focusing on universal health coverage with essential public health interventions, including access to preventive chemotherapy (PC) for NTDs. The medicines used in PC for LF, onchocerciasis, soil-transmitted helminthiases (STH), schistosomiasis and trachoma are all safe, single dose medicines that can be used without prior individual diagnosis. The rationale for expanding integrated PC is that the same individuals in poor environments are affected by multiple diseases and programmes have an ethical responsibility to treat all diseases. Integrating and coordinating PC interventions can result in increased cost-effectiveness, feasibility, visibility, acceptability, and can reduce the risk of drug resistance. Given that 1.4 billion people require PC for LF and that LF drug distribution is community based, LF is often used as the platform for integrating PC diseases. Other strategic components overlap among the disease programmes, such as morbidity management (LF, schistosomiasis, trachoma), vector control (LF, onchocerciasis, schistosomiasis) and water and sanitation (schistosomiasis, STH, trachoma).

He noted that the goal of integrated PC is to eliminate LF, onchocerciasis, blinding trachoma and in some cases schistosomiasis as public health problems and eliminate childhood morbidity related to soil-transmitted helminthiases by 2020. This will be achieved by scaling up PC for all diseases to full national scale as quickly as possible, and enhancing these interventions through integrated planning, implementation and management. Steps for rolling out integrated PC include: i) institutionalizing a NTD task force, ii) developing a national plan of action through situational analysis, action planning and mapping, and identifying funding gaps, and iii) holding a stakeholders meeting to mobilize resources and coordinate partners.

**Figure 1:**
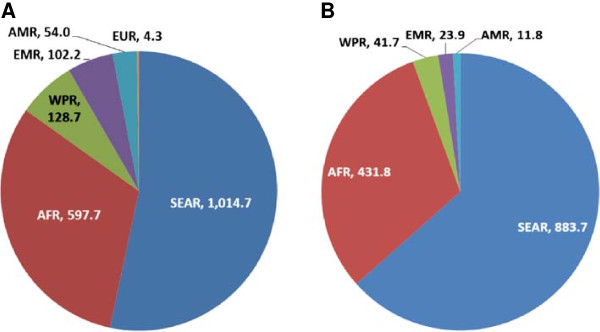
**Overlap between population requiring PC for all diseases and population requiring MDA for LF.** Legend: **A**: Population requiring PC 1.9 billion. **B**: Population requiring MDA for LF 1.39 billion. AFR: Africa Region, AMR: Americas Region, EMR: Eastern Mediterranean Region; EUR: Europe Region, SEAR: South East Asia Region; WPR: Western Pacific Region.

Currently, 1.9 billion people in 124 countries require PC for at least one of the diseases, with 710 million treated in 2010. The WHO South-East Asia Region has 53% of the burden and the African Region has 31%. Specifically for LF, 1.4 billion people in 73 countries require MDA, with 539 million treated in 2010–2011. The South-East Asia Region has 63% of the global burden of LF, while the Africa Region has 31%. Thus successful scale up for LF MDA can also cover the majority of the global burden of PC (Figure 1).

However, Dr Engels cautioned that there is still a need to sustain the impact of LF MDA through non-LF PC once the LF programme scales down, particularly for STH and onchocerciasis. While there often will be a shift to school-based PC after LF MDA ends in some areas, some programmes will still need community-based interventions.

## LF strategic plan

Dr Kazuyo Ichimori (WHO Department of Control of NTDs, Geneva) presented the current status of the Global Programme to Eliminate Lymphatic Filariasis (GPELF) as 73 countries^a^ endemic for LF, 1.39 billion people at risk, 120 million infected, and 40 million people affected by morbidity. The goal of global elimination has two parts: i) to stop the spread of infection and interrupt transmission through MDA and ii) to reduce human suffering through managing morbidity and preventing disability (MMDP). In 2011, 53 countries implemented MDA, with 538.6 million people treated in 2010–2011, and 12 countries under post-MDA surveillance. However, only 27 countries reported MMDP activities to WHO. Baseline sentinel site data from 37 countries showed an average microfilaraemia prevalence of 12.6%, which dropped to 2.7% according to mid-term sentinel site data from 36 countries.

**Figure 2:**
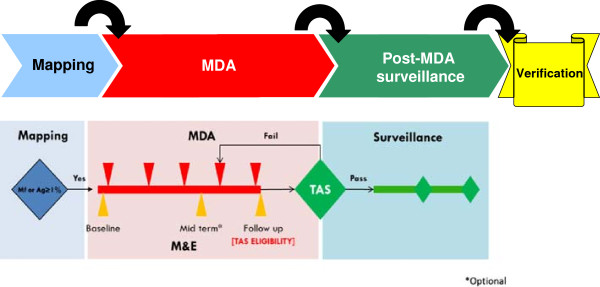
Programmatic steps: MDA component.

Dr Ichimori then summarized the GPELF Strategic Plan 2010–2020 [1], which was developed largely as a result of conversations at the last GAELF meeting in Korea [2]. The vision of the strategic plan is a world without the risk of LF through global elimination of LF as a public-health problem. The objective for the MDA component is to reduce infection prevalence below target thresholds in all endemic areas, with targeted prevalence levels defined for various species/vector complexes. This is achieved through stepwise activities of mapping, MDA, post-MDA surveillance, and verification (Figure 2).

In order to achieve the MDA goal, the strategic plan included four milestones for 2011–2013: i) revised monitoring and evaluation (M&E) guidance, ii) criteria for verifying absence of transmission, iii) provisional strategy for interrupting transmission in loiasis-endemic areas, and iv) final strategy for interrupting transmission in loiasis-endemic areas. The M&E manual, which includes guidance on verifying absence of transmission, has been published [3]. The provisional strategy for loiasis-endemic areas has been recommended to the Strategic and Technical Advisory Group for NTDs for endorsement in April 2013 [4]. The MDA target is that by 2020 70% of countries will be verified free of LF and the other 30% will be under post-MDA surveillance. As of 2011, 19 countries had not started MDA, 41 were implementing MDA, 12 were under post-MDA surveillance, and nine had been verified free of LF.

**Figure 3:**
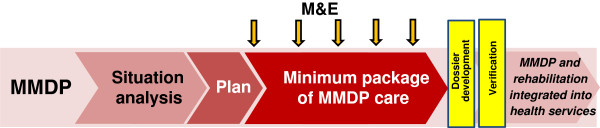
Programmatic steps: MMDP component.

The target for the MMDP component is no new clinical cases, measured by i) reduction in the number of acute attacks, ii) the number of hydrocele patients cured, and iii) the MMDP geographical coverage in LF-endemic countries. This is achieved through analyzing the MMDP situation in country, developing a plan, and providing a minimum package of MMDP care (Figure 3).

In order to achieve the MMDP goal, the strategic plan included two milestones for 2011–2013: i) revised guidelines and training modules for MMDP, and ii) metrics developed for annual reporting on MMDP activities to WHO. A position statement has been published [5] and the MMDP manual for programme managers, which includes indicators and forms for reporting to WHO, is currently in draft. The MMDP target is that by 2020 100% of endemic countries will have achieved full geographical coverage and access to basic MMDP care.

**Figure 4:**
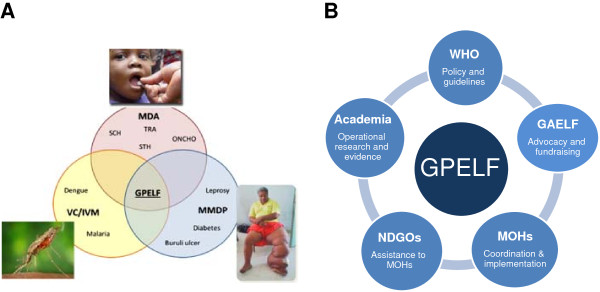
**Working together to meet the GPELF goal.** Legend: **A**: Integration: MMDP, vector control and MDA. **B**: Partnership: Collaboration to support one GPELF. MDA: mass drug administration; SCH: schistosomiasis; STH: soil-transmitted helminthiases; TRA: trachoma; ONCHO: onchocerciasis; VC: vector control; IVM: integrated vector management; GPELF: Global Programme to Eliminate Lymphatic Filariasis; MMDP: morbidity management and disability prevention; GAELF: Global Alliance to Eliminate Lymphatic Filariasis; NGDO: non-governmental development organization; MOH: ministry of health.

Critical challenges include: increasing LF MDA coverage to 100% while coordinating with other PC diseases and vector control; planning for TAS; ensuring availability and affordability of diagnostic tests; developing effective post-MDA surveillance tools; defining the role of the regional programme review groups and the Strategic and Technical Advisory Group for NTDs in the verification process; scaling up MMDP activities; and collecting evidence on best practices of linking MMDP activities with health services. These challenges can be best met by using a network of partners and integrated approaches (Figure 4) to work on policy, advocacy, implementation, and operational research.

## Regional progress

**Figure 5:**
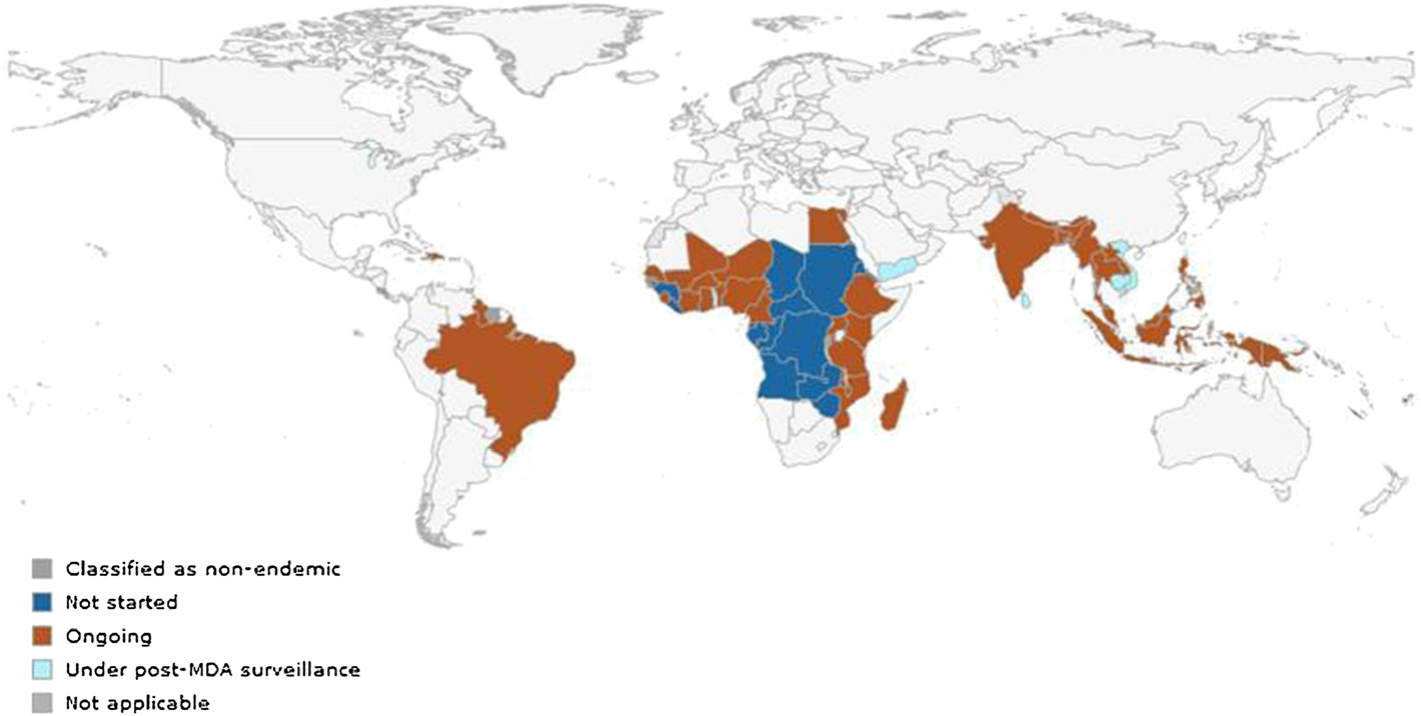
Status of MDA by country.

Dr Ricardo Thompson (National Institute of Health, Mozambique) presented the status of the Programme to Eliminate LF in each of the WHO Regions: African Region, Americas Region, Eastern Mediterranean Region, South-East Asia Region, and the Western Pacific Region. Despite success in scaling up MDA, regions face challenges as they reach the peak of delivering required treatments, while simultaneously needing to build capacity to implement TAS to evaluate success. He presented regional statistics on the number of countries and people requiring MDA, the number treated, and country status (Table 1 and Figure 5).

**Table 1:** Number of countries and people requiring MDA and number treated, by WHO Region

**Region**	**No. of countries requiring LF MDA**	**Total population (m) requiring MDA**	**No. people (m) treated in 2011**
Africa	34	443	113
Americas	4	12	9
Eastern Mediterranean	4	22	0.5
South-East Asia	9	879	414
Western Pacific	22	37	21
TOTAL	73	1,393	557.5

### African Region

The African Region has achieved remarkable progress in scaling up effective MDA since 2000, with 76% coverage of total population in areas with MDA in 2011. However, by 2015 both the number of countries conducting MDA and the number treated must be doubled. In particular, the countries which have not finished mapping or started MDA, mostly in Central Africa, must start, especially now that guidelines for MDA in loiasis-endemic areas are available. The region faces logistical and political challenges which have delayed completion of mapping and initiation of MDA. Heavy burden countries such as the Democratic Republic of Congo, Nigeria and Ethiopia need specific plans to aid in scaling up, including consideration of alternatives to annual MDA to accelerate the progress of interruption of transmission. The regional partnership is being strengthened to support country implementation.

### Americas Region

The high burden of LF in Haiti, which constitutes 80% of the Americas burden, presents a special situation, especially after the earthquake in 2010 which destroyed much of the government’s infrastructure. However, LF MDA in 2012 achieved full national coverage for the first time helped, in part, by the strong regional partnership that includes Ministries of Health and Education, IMA World Health, University of Notre Dame, US Centers for Disease Control and Prevention (CDC), United States Agency for International Development (USAID), Inter-American Development Bank, and the Global Network for NTDs. In addition, a bi-national project to eliminate malaria in Haiti and the Dominican Republic will also have positive effects for the LF programme.

### Eastern Mediterranean Region

The Eastern Mediterranean Region showed a peak in treatments in 2004, with numbers of people treated drastically falling off from 2005 onwards, since two of the LF-endemic countries are moving towards elimination and scaling down treatments, while the fragile infrastructure in Sudan and South Sudan has made it difficult to upscale MDA. Yemen and Egypt now need to procure immunochromatographic tests (ICTs) to carry out TAS since all implementation units (IUs) have implemented at least eight rounds of MDA and achieved <1% microfilaraemia rates in sentinel and spot-check sites. In addition, the region needs to strengthen morbidity management.

### South-East Asia Region

All the LF-endemic countries in the South-East Asia Region are implementing MDA, except Sri Lanka and Maldives which are under post-MDA surveillance. While the high-burden countries of Bangladesh and India have made significant progress in implementing MDA, they are facing difficulties procuring ICTs for implementing TAS. Political instability in Timor Leste led to the interruption of MDA, which has not restarted. The region also must strengthen the commitment of implementation partners in the region and scale up MMDP activities.

### Western Pacific Region

In the Western Pacific Region, three countries have not started MDA, nine have ongoing MDA and ten are under post-MDA surveillance. Challenges include availability and affordability of ICTs, reaching prevalence goals in areas with *Aedes* vectors where repeated MDA rounds have not lowered levels adequately, and strengthening MMDP activities. Scaling up MDA in Papua New Guinea (PNG) continues to be a major task. Finally, there is a lack of country-level capacity to prepare dossiers for verification, which will become a challenge in other regions as countries progress.

## Discussion

In order to truly analyze regional progress, participants suggested that presenters provide details of the epidemiological situations and implementation progress at an IU level. In addition, the most appropriate way to provide support to countries – given the multiple demands on WHO and regional programme review group members - was debated, with a suggestion to allow decisions by a country-level review group under the auspices of the regional programme review groups for larger countries. Given the need to build country and regional capacity to analyze country situations and review programme progress, participants were asked to give specific ideas on short- and medium-term capacity building needs to Professor David Molyneux, who is chair of the WHO NTD working group on capacity building. Finally, an update on the new ICT platform was provided: it is currently undergoing field evaluation, and the effect of the new test format on TAS guidance will be reviewed after the results are compiled. A small group of partners is in discussion with Alere, the company that produces the ICTs who have requested accurate demand forecasts from country programmes in order to ensure supplies are adequate and timely.

### Highlighting success

#### Highlighting success in scaling up

Moderator: Mr Andy Wright (GlaxoSmithKline, UK).

##### Haiti: triumph over adversity

Dr Abdel Direny (IMA World Health, Haiti) described the LF situation in Haiti, where 88% of communes have *Bancroftian* filariasis, transmitted by *Culex* mosquitoes. MDA began in 2000 in the Leogane commune and scaled up to 24 communes by 2005, when it was stopped due to lack of funding. Since 2008, with help from GSK, IMA World Health/USAID, University of Notre Dame, CDC, and Partners in Health, the programme has gradually scaled up, reaching full coverage of 140 communes in 2012. Coverage in the 2011–2012 round of MDA averaged 90% of total population, with a range between 80-116% (using population numbers based on 2003 census data without accounting for migration). Drugs are administered at distribution posts and schools, with three community drug distributors per post. Social mobilization is accomplished through radio spots, announcements from trucks, and community leader announcements. The community drug distribution networks are also used to distribute shoes, water filters, and essential medicines.

Dr Direny recounted the many challenges of the Haiti programme, including the 2010 earthquake, cholera outbreaks, and political instability. The programme needs to scale up morbidity management, latrine construction, hygiene activities, and determine how to continue with STH MDA after LF MDA is stopped. He emphasized that these challenges can only be addressed through collaboration; integration of LF and STH programmes; and participation of community leaders, health promoters and community drug distributors in population sensitization to ensure sustained interest in participating in MDA. The triumph of the programme over unavoidable adverse situations was feasible due to the commitment of government and partners, the ability to immediately assess and plan next steps after the earthquake, the contributions from volunteers, and the consistent use of monitoring and evaluation to measure impact.

##### Malawi: an inspirational personal story

Mr Square Mkwanda (LF Programme in the Ministry of Health, Malawi) discussed the history of the LF programme in Malawi, where 26 of 28 districts are endemic, with 14.1 million people at risk. While the country was mapped in 2003, MDA did not start until 2008, after the Minister of Health was influenced by hearing about other countries’ progress at the 2008 GAELF meeting in Tanzania. Even though there was no LF-specific budget, Mr Mkwanda was tasked with rolling out LF MDA in eight districts, which he achieved through integration with other health activities, such as distribution of bednets. In order to show political will and convince the public the drugs were safe, drugs were distributed in parliament and the state house. After achieving 80% coverage, the programme scaled up in 2009 to total geographical coverage, and was able to use the results of 2008 and 2009 MDA to secure support from the Centre for Neglected Tropical Diseases (CNTD) for 2010 onwards. He noted that challenges to eliminate remain, including cross-border issues, morbidity management, and competing government priorities. Although morbidity management is ongoing, with 3,201 hydrocelectomies and 2,008 lymphoedema patients treated over the past four years, there are gaps in supervising self-care groups and building capacity of health workers in case management.

##### India: impact of government commitment

Dr V Kumaraswami (Consultant, India) presented the status of LF in India, which accounts for 40% of the global burden with 600 million people at risk and 509 million targeted for MDA in 20 endemic states. MDA for LF was piloted in the 1950s, with a 2002 national health policy calling for elimination by 2015. Single-dose DEC MDA covered all endemic areas by 2004 and DEC + albendazole MDA covered all areas by 2006; however, issues of social mobilization and compliance still exist. Sentinel sites show a positive impact of MDA – with an average 1.2% mf rate in 2004, compared to an average of 0.35% in 2011.

In 2011, the programme was supported by US$10 million for MDA and morbidity activities from the central government, state budget contributions, and a donation of 300 million albendazole tablets by GSK. Other partners include medical colleges to implement independent coverage assessments, the Indian Council of Medical Research to assess MDA coverage and implement operational research, the National Centre for Disease Control to train, and non-governmental development organizations to implement and evaluate morbidity activities.

He noted some of the complexities of the Indian programme, including drug needs (1 billion DEC tablets and 500 million albendazole tablets a year), quality-assured drug procurement, storage and distribution of drugs, training of 2.5 million peripheral health workers, and treatment of 250 people per day per worker during MDA. Current critical issues include scaling up morbidity training, hydrocele surgery, and home-based lymphoedema care, as well as strategies and funding for scaling down, as 170 IUs are ready for TAS, necessitating procurement of 150,000 ICTs in 2013 when TAS activities are conducted.

##### Liberia: capitalizing on existing platforms

Dr Karsor Kollie (LF Programme, Ministry of Health, Liberia) described Liberia’s LF programme, which covers the 13 endemic counties and an at-risk population of 3.4 million, in a country that is post-conflict, with a weak health system. The LF programme built on the success of the onchocerciasis programme, which uses community drug distributors (CDDs) for MDA and has a mechanism in place to monitor the impact of MDA. The programme targeted 1.6 million people in the 13 endemic counties in 2012, with an aim of 100% coverage in the next two years. The largest challenges have been the turnover of CDDs due to poor motivation and logistical issues, such as the need for motorbikes to reach isolated areas. Integration with the malaria programme has been helpful to determine the impact of vector control on MDA and determine the existence of urban LF transmission. Lessons learned included: integration can enhance coordination and outcomes, use of existing structures increases ability to scale up, and collaboration with malaria programmes on integrated vector management might speed up LF elimination.

Support for the programme is provided by CNTD, Sightsavers, WHO and the African Programme for Onchocerciasis Control (APOC). In addition, LF is now included in the 10-year national health policy and essential package of health services, so government funding will hopefully increase. Continued government advocacy will be important, especially as the President of Liberia is now co-chairman of the United Nations committee shaping the post-Millennium Development Goals priorities.

#### Highlighting success in scaling down

Moderator: Dr Adrian Hopkins (Mectizan Donation Program, USA).

##### Togo: a first in Africa

Dr Monique Dorkenoo (LF Programme, Ministry of Health, Togo) discussed how Togo – the only country in Africa that has been able to stop MDA – was able to scale down. Togo has 1.3 million people at risk of LF in seven districts. MDA started in one district in 2000, with full geographic scale achieved in 2003 and continued for at least six years in all districts until 2009. Every district had a sentinel site with regular evaluation of microfilaremia levels. In 2008, all districts were assessed for stopping MDA using the 2005 WHO guidelines. MDA was stopped in five of seven districts after the 2008 MDA and in two districts after the 2009 MDA. She noted that the critical challenge is how to monitor for, and avoid, resurgence. A first round of post-MDA surveillance using TAS was implemented in Kozah district in December 2009 as part of the BMGF-supported global operational research project and a second round will be implemented in 2012. The programme aims to maintain advocacy to ensure funding for post-MDA ongoing surveillance, currently focused in border areas. It also is working to increase bednet coverage and usage to ensure interruption of transmission.

##### Ghana: gradually scaling up and down

Dr Nana-Kwadwo Biritwum (NTD Programme, Ministry of Health, Ghana) described how LF activities in Ghana are administered in the context of the NTD programme, as every district in Ghana is endemic for at least three NTDs. The population at risk of LF in Ghana is 12 million, in 74 of 170 districts. From 2001–2006, Ghana scaled up MDA from five districts to all 74 endemic districts. Coverage surveys were completed in three to ten districts each year. As of 2010, four districts have stopped MDA. He noted that the three greatest challenges were i) urban MDA and community fatigue in rural areas, ii) tools for post-MDA and non-endemic district surveillance, and iii) limited human and financial resources. In order to overcome these challenges, the programme is working to develop alternative social mobilization and distribution strategies for urban MDAs, implementing operational research with CDC on surveillance, and building technical capacity.

##### Vietnam: planning for dossier development

Dr Do Trung Dung (LF Programme, Ministry of Health, Vietnam) described LF in Vietnam, where both *Bancroftian* and *Brugian* filariasis are present. One and a half million people are at risk in 12 districts, which were combined into six IUs for MDA implementation. From 2003–2008, all six IUs underwent five rounds of MDA. In 2009, all districts were assessed for stopping MDA using the 2005 WHO guidelines. Post-MDA TAS surveys will be implemented in 2013 and in 2015, grouping districts into four evaluation units. He noted that a major challenge is to conduct TAS in schools in rural, minority areas due to difficulties obtaining consent for blood collection. The programme aims to involve teachers and parents with survey teams when collecting blood in these areas. In addition, the programme will need external financial and technical support, as it has limited government funding and limited ability to collect data and technically write the verification dossier.

##### Philippines: ensuring government support at all levels

Dr Leda Hernandez (Ministry of Health, Philippines) explained that 28–30 million people are at risk of LF in the Philippines in 44 endemic provinces. MDA has been ongoing since 2001 in selected areas, with a peak of 37 IUs conducting MDA in 2007 and 2009. As of 2011, 12 provinces stopped MDA, with TAS scheduled for eight more in 2012. Three IUs have finished post-MDA surveillance, and six others have ongoing surveillance. She noted that the key drivers of this success have been government budget (e.g. for DEC), partnerships to fill gaps and address morbidity, presence of committed health workers, and external technical assistance. The programme is addressing the challenge of ownership and commitment of local government units by offering performance-based incentives for IUs which achieve elimination goals. It also is holding regular stakeholder meetings to increase coordination of, and feedback to, partners, especially those from other sectors; training health workers on new guidelines; and conducting rapid assessment surveys, operational research, and post-MDA surveillance in order to strengthen M&E. Finally, the programme is lobbying for the sustainability of the annual LF budget until the elimination goal is reached –not just until MDA is stopped.

## Discussion

Four main issues were discussed following these presentations. First, the importance of collecting data on baseline prevalence was stressed, as it is a factor in determining how many rounds of MDA are necessary. Second, the challenges of cross-border collaboration and synchronizing MDA cycles were brought up, with a suggestion to intensify surveillance in cross-border areas. Third, the suggestion of twice-yearly treatment was put forward to speed interruption of transmission, especially in places with twice-yearly onchocerciasis treatment. Finally, it was suggested that it would be helpful if countries found a way to acknowledge donors and partners to encourage sustained donations in a tough financial climate.

### Meeting programme challenges

#### Reaching the unreached

Moderator: Professor Moses Bockarie (Center for Neglected Tropical Diseases, UK).

##### Sierra Leone: how did Sierra Leone do it?

Mr Mustapha Sonnie (Helen Keller International, Sierra Leone) described the LF situation in Sierra Leone, where LF is co-endemic with onchocerciasis and transmitted by the *Anopheles* vector. Despite prior conflict, a poor health system, and low technical capacity, the programme is on track to eliminate LF transmission by 2015. Building on the well-established community-based onchocerciasis MDA system, the LF MDA was scaled up to national coverage in the three years from 2007 to 2010. Training and advocacy follow a cascade from national supervisors to district health management teams to primary health unit staff to community drug distributors, with targeted messages for each. Social mobilization activities include community meetings, interactive programmes in urban areas, and radio programmes.

Mr Sonnie noted that the programme faces challenges of competing health priorities such as cholera, high infant and maternal mortality and malaria; poor road infrastructure; and motivation of community volunteers. Community leaders are asked to find ways to motivate volunteers, such as exemption from communal work, helping CDDs with farming activities during the harvest season, and recognition by the community. The national NTD programme and partners have over the years provided motivation to volunteers such as free t-shirts and certificates. In addition, traditional beliefs that equate elephantiasis with witchcraft mean that the programme has to reach out to traditional healers to ensure their buy-in to the MDA. Partners include USAID, DFID, Helen Keller International, Sightsavers, and CNTD.

##### Liberia: why is Liberia so optimistic?

Ms Marnijina G. Moore (Marjin) (LF Programme, Ministry of Health, Liberia) described Liberia’s plan to scale up to 100% geographic coverage of LF MDA in the next two years. The programme’s strategy relies on an integrated programme with onchocerciasis, STH, schistosomiasis, Guinea worm and Buruli ulcer, led by a single director and a motivated young team. MDA for LF and STH are being added to the onchocerciasis community-directed treatment intervention platform, which covers the entire country. Success is helped by the relatively small size of the country of 3.4 million people, and a decentralized health system that enables counties to plan and implement their own NTD interventions. Commitment from the government of Liberia and donors such as APOC, DFID, CNTD and Sightsavers allowed the programme to add more trained health workers and CDDs after LF was added to the onchocerciasis platform. She remarked that even though people might not understand the details of the NTD strategy, there is a demand for ivermectin from communities which made it easy to add albendazole to the MDA. Challenges of infrastructure, motivation of volunteers, vertical programmes, a limited government budget, poor inter-sectoral collaboration, and lack of a mechanism for morbidity management were noted.

##### Papua New Guinea: why is PNG struggling?

Dr Leo Makita (Malaria and Vector Borne Diseases, Ministry of Health, PNG) discussed the LF situation in PNG, where LF is endemic in most districts and transmitted by the *Anopheles* vector. Seventy-five percent of the population lives in rural areas. He noted that PNG’s challenge to find enough resources to deliver health services to all areas remains unmet. While the LF programme achieved success at scaling up MDA in 2004 by integrating with malaria programme bednet distribution, in the following years there were not enough resources to implement LF MDA alone.

Using the health system to reach those at risk has produced limited results, due to areas that have no access to any health services (where health posts might be closed due to tribal fights), a decentralized system where civil servants in each district have to be convinced to implement MDA, the high cost of MDA, the length needed to complete an MDA using health staff, and unsatisfactory reporting. In addition, finding health staff to supervise volunteer distributors, drug logistics, and developing social mobilization strategies for many unique communities has proven difficult. Other health programmes, such as malaria, have found success using private partners to implement health activities, such as Rotary’s attainment of 95% national coverage of bednets in a short time frame.

Dr Makita summarized PNG’s plan to move forward by grouping villages into manageable populations for MDA, appointing and training more volunteers, having MDA on an appointed day in a district, recruiting extra national staff, and ensuring availability of drugs and adequate information. The new PNG Minister of Health has renewed the government commitment after attending a NTD meeting in Australia, and partners such as WHO, James Cook University, the Institute of Medical Research, and the private sector are becoming more involved.

## Discussion

Participants offered suggestions for the PNG programme, including a cost comparison of using community volunteers versus hiring a third party to implement, piggybacking on the Expanded Programme on Immunization activities, looking at cross-border solutions with Indonesia, and exploring private sector infrastructure networks such as those distributing cell phones, liquor, etc.

### How can we accelerate the process?

Moderator: Dr Frank Richards (The Carter Center, USA).

### Indonesia: moving from partial to full coverage

Dr Rita Kusriastuti (Vector Borne Disease Control, Ministry of Health, Indonesia) summarized the LF situation, where 368 of 497 districts are endemic, with 125 million people at risk of *Bancroftian* and/or *Brugian* filariasis. The programme defines the district as the IU, but many districts are only able to support MDA in some sub-districts each year. In 2011, 98 districts conducted MDA, with 61 covering the entire district and 37 covering only some sub-districts. In addition, some districts have conducted more than five MDA rounds, but are still endemic. These issues have led to problems in fully implementing the elimination strategy, which called for MDA implementation in 80 districts per year from 2012–2018. To accelerate progress the programme needs to improve programme management by strengthening case finding, reporting and recording; involve the community in the population registration; increase monitoring of ingestion of the drugs; and improve the referral system for serious adverse reactions. In addition, human resource capabilities need to be improved through training in planning and surveillance, managerial skills, communication skills, and monitoring and evaluation.

Dr Kusriastuti noted that expansion of the national LF committee to cover NTDs, advocacy to both high-level leaders and local government leaders to support operational costs, integration with the school health programme for STH, partnerships with other sectors, involvement of local non-governmental development organizations, and assistance from international partners such as WHO, USAID, AusAID, RTI International, Rotary, World Vision, and the Global Network for NTDs is all critical to achieve the elimination goal. Although $12 million is already committed by donors and the government, the Indonesia programme has a funding gap of $10 million in 2013.

### Nigeria: improving integration to assure funding

Mr Chukwu Okoronkwo (NTD Programme, Ministry of Health, Nigeria) explained that over 106 million people are at risk of LF in 541 local government units (LGAs) of the 705 that have been mapped. Only 175 LGAs (32%) implemented MDA in 2011, covering an at-risk population of 33 million, but with less than half of the LGAs achieving the coverage goal of 80% of eligible population treated. The programme started scaling up MDA in 2009 in areas co-endemic with onchocerciasis, with 4.7 million treated in 2009, 10 million treated in 2010 and 22 million treated in 2011. He noted that the programme intends to complete mapping by March 2013 with USAID and DFID support, conduct baseline surveys in sentinel sites, provide adequate management and technical training to improve planning and coordination skills of State Coordinators, and conduct comprehensive census updates. However, plans to accelerate progress have been hampered by ineffective coverage of MDA in urban areas, which has brought overall coverage rates down. Financial issues of inadequate funding, late release of approved funds by partners, and dependency on assisting non-governmental development organizations can be solved by advocacy and sensitization at the highest levels, improving the integrated NTD platform implementation to share resources, and mobilizing additional partners. The programme also needs to address the issue of incentives for CDDs and health workers, as funds are insufficient. Policymakers’ desire to extend MDA to areas that are not yet mapped or are non-endemic have been problematic, so the programme aims to target more sensitization and advocacy activities to policymakers at all levels.

### Tanzania: phasing in integrated MDA

Dr Upendo Mwingira (National NTD Programme, Ministry of Health, Tanzania) discussed the integrated NTD programme on mainland Tanzania, which includes five PC NTDs and ten case-management NTDs, targeting 44 million people. The programme follows a NTD master strategic plan, implemented by NTD coordination units at national, regional and district levels. It uses the LF MDA as the platform, training health workers, school teachers and CDDs, and is scaling up MDA through a phased approach of adding one region each year. In 2011, 11.3 million people were treated for NTDs in 76 implementation units (50% geographic coverage), with the goal to reach full geographic coverage by 2014. She noted that the programme needs a NTD database system that can send data collected at health center level directly to national level and can be integrated into the health management information system in order to overcome challenges of delays in data collection and reporting. The programme will encourage increased programme monitoring, using partner support to maintain at least one sentinel sites in every region and will increase advocacy and support for morbidity management at all levels. Other technical issues include the need for clear tools for all NTDs, more training, and support for a drug logistician. In terms of administrative issues, Dr Mwingira remarked that an integrated country strategic plan and joint planning meetings are necessary when dealing with multiple partners and donors, including CNTD, the Schistosomiasis Control Initiative, RTI International, IMA World Health, Sightsavers, and Helen Keller International. Local advocacy is necessary to ensure local government contribution and engage partners from other sectors. The programme sees opportunities in a strong health system, existing partnerships, links to interventions such as Water, sanitation and hygiene (WASH), indoor residual spraying for malaria, and the global momentum resulting from the London Declaration.

## Discussion

Participants remarked on the tension between scaling up and ensuring appropriate treatment coverage in current MDA areas, advising that it is important to meet treatment coverage in current areas first before expanding geographic coverage. Suggestions on improving advocacy and funding included the use of ministers of parliament and involving African and Asian Development Banks. Decentralized systems that have power at district level can help accelerate progress as services are delivered nearer the community, freeing the central level to focus on advocacy and coordination. The issue of finding hot spots, e.g., clusters of residual transmission, in large districts was also raised.

### Strategies for achieving disability prevention and morbidity management

Moderator: Ms Ann Varghese (IMA World Health, US).

#### Togo national programme for lymphoedema management

Dr Dorkenoo summarized the three elements of the national lymphoedema management programme: detection and referral of cases, treatment and management of cases, and follow up and evaluation. In every village, village chiefs and community leaders are asked to inform the communities that anyone with a large swollen leg should go to a clinic and this is enforced through TV and radio announcements, posters and public criers. A national training-of-trainers model trains nurses from throughout the country and community health workers in seven endemic districts. The national programme does periodic supervision of nurses and patient visits.

She described how the programme started in June 2007 in the seven endemic districts and was scaled up within two years to the entire country. Even though LF MDA ended in 2009, the number of patients continues to increase, with over 1,200 patients currently under treatment, and 32% of patients living in non-endemic districts where LF MDA was not needed. Operational research funds to determine the feasibility of a countrywide morbidity control programme included start-up funding for training, IEC material, washing kits and supervision. The well-functioning and decentralized health system is a good platform for the programme, with the aim to continue providing services even after the LF elimination target of 2015. The Ministry of Health continues to maintain the LF programme coordination staff posts, even though MDA has stopped, and provides support for supervision activities. However, Dr Dorkenoo noted challenges with a high turnover of trained staff and long-term compliance of patients, necessitating refresher training and continuing behavior change communication activities.

#### Community-based support for lymphoedema patients

Mr Jonathan Rout (Churches Auxiliary for Social Action, India) described their project to provide community-based support for lymphoedema patients in the highly-endemic state of Orissa, where 3.5 to 6.3 million people are estimated to be infected with LF. The multi-faceted approach includes health education to entire communities, patient registration, extensive training, patient follow up, and impact assessment. In a census of 215,515 households, 27,671 people with hand swelling, breast swelling, hydrocele or lymphoedema were found. At least one LF task force volunteer per village was trained and certified in lymphoedema management and basic first aid and, in turn, distributed posters and leaflets door-to-door. They continue to follow closely 20 patients each. The project also supports wall paintings and street plays to raise awareness of MDA and footcare management, and implements classroom and field training in lymphoedema management for school children, family members, village volunteers, and paramedics. Patients are provided with soap, anti-fungal cream, towels, shoes, and a 365-day booklet to track exercises and routine hygiene. An impact study found that quality of life improved significantly among patients, with daily washing associated with lower disability scores. The frequency of acute attacks was the strongest predictor of disability. In addition, a CDC study found that MDA coverage in districts with the lymphoedema management programme (90.2%) was better than in districts with MDA only (52-59%) or a community-based pre-MDA education programme only (75%) [6].

#### New therapeutic options for lymphoedema

Dr Achim Hoerauf (University of Bonn, Germany) presented the results of recent research that aimed to determine whether doxycycline improves filarial lymphoedema in filarial antigen-positive patients (e.g., those with ongoing infection) as well as antigen-negative patients [7]. In addition, it compared the effect of doxycycline, an antibiotic which targets *Wolbachia* as well as exogenous bacteria, to amoxicillin, an antibiotic which targets exogenous bacteria only. The study had three treatment arms: i) doxycycline 200 mg/day for 6 weeks and hygiene management, ii) amoxicillin 1000 mg/day for 6 weeks and hygiene management, and iii) placebo drug and hygiene management. Approximately 40 patients were in each arm, the majority of whom were in stages 1–3. Dr Hoerauf presented results that doxycycline was beneficial in reverting or halting progression of early stage lymphoedema, regardless of whether patients had active infection. The results suggest that doxycycline might have an anti-inflammatory effect that makes it superior to other antibiotics. Of those patients treated with doxycycline, 37% showed improvement in stage, compared to 3.2% of those treated with amoxicillin and 5.6% of those treated with placebo. A lack of progress to more severe stages was shown in 95% of those treated with doxycycline, 71% with amoxicillin, and 44% with placebo. There is a need to extend this research to patients with stages 4 and higher, as well as to replicate it in other settings. The study recommended that a 6-week course of doxycycline be given to lymphoedema patients once every two years as part of morbidity management.

#### African LF morbidity project

Dr Sunny Mante (African LF Morbidity Project, Ghana) explained its goal to build capacity for filarial hydrocele surgery, reconstructive plastic surgery for genital lymphoedema, and hygiene therapy for lymphoedema. A 2006 evaluation of the BMGF-supported pilot project to train hydrocele surgeons reached 399 of the 1,771 patients who had received hydrocelectomies and found that 92% were very satisfied with the surgery. Many patients were lost to follow up because they had gone back to work or were away for seasonal agriculture.

The project was scaled up in 10 West African countries plus Tanzania, and Malawi through funding from Health and Development International and International Volunteers in Urology, training 469 people in 12 countries in hydrocele surgery and operating on 3,975 patients during training. A 2007 revised surgical manual details the technique used, which includes total removal of the sac and no drains [8]. Since 2011, training on hernia surgery has been explicitly included in these trainings, as it is difficult to differentiate between hydrocele and hernia patients pre-operatively and many hernia patients present for surgery when free hydrocele surgery is offered.

Dr Mante noted that currently four workshops are planned (two each in Ghana and Sierra Leone) through support from International Volunteers in Urology and Johnson & Johnson, although the project would like to extend to East Africa and Asia. Because 25-40% of adult males have hydrocele in highly LF-endemic areas, there is a need to scale up provision of surgeries, provide support to hernia patients, report and evaluate surgeries ongoing in all countries, and ensure hydrocele surgery techniques are included in routine medicine training in endemic countries. Capacity building of plastic surgeons on reconstructive surgery for genital lymphoedema is also needed. Finally, while two articles on hydrocele surgery were recently published [9,10], there is a need for more advocacy and publishing of results.

## Discussion

Integration of these activities with other diseases was the major topic of discussion. The African LF Morbidity Project is in discussion to have joint workshops for hydrocele, genital lymphoedema, and urogenital fistula, although it was noted that it might be difficult to combine trainings of district surgeons responsible for hydrocele with gynecologists who are responsible for urogenital fistula. Treating podoconiasis patients in conjunction with lymphoedema patients was seen as useful, given that management is similar. In addition, the importance of having countries report on these activities to WHO and collecting data from the hospital system on number of operations, complication rate, and recurrence was raised.

### Linking the programmes

#### New partnerships with onchocerciasis

**Figure 6:**
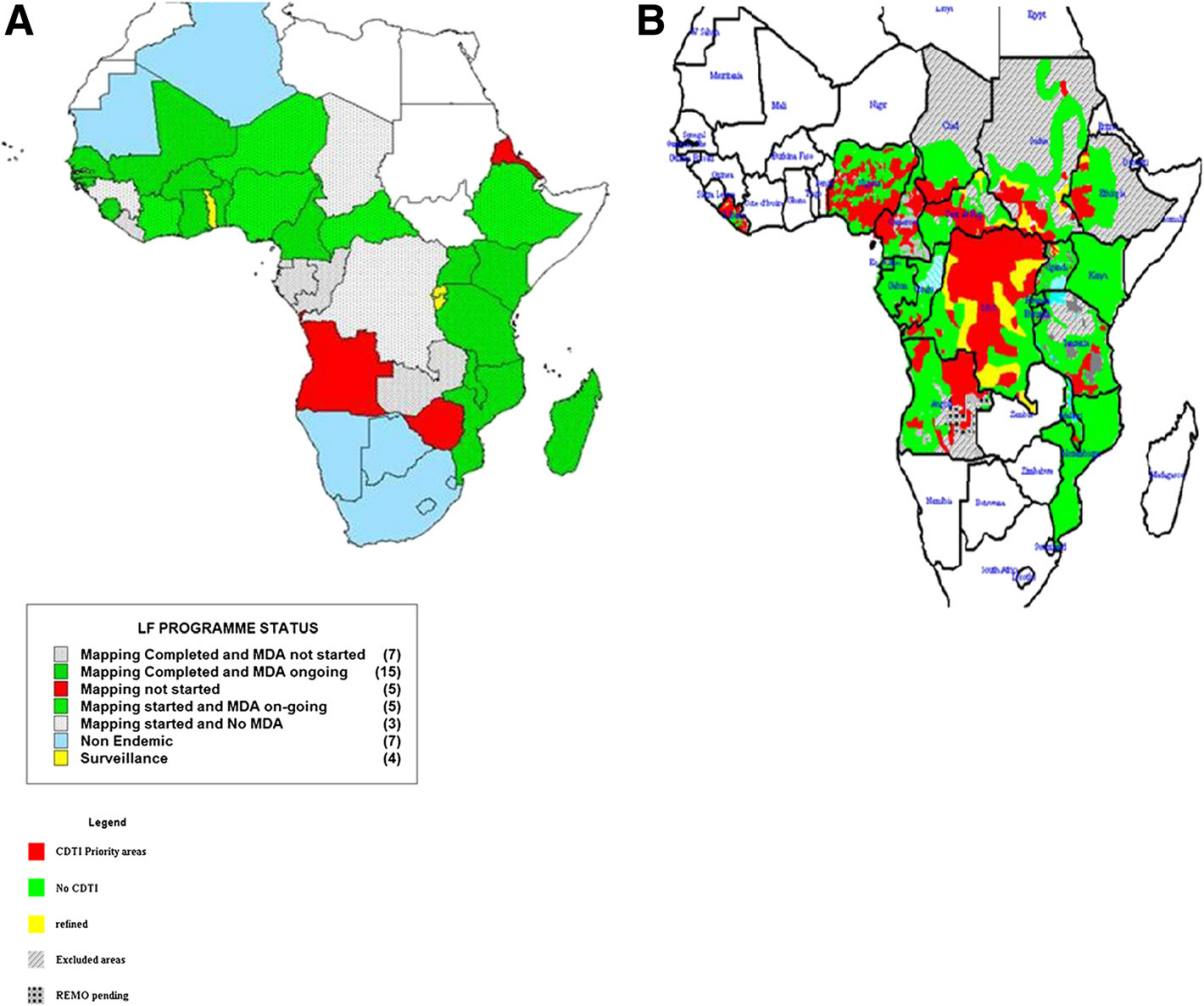
**Overlap between LF and onchocerciasis in Africa.** Legend: **A**: Lymphatic Filariasis current status (Source: AFRO NTD programme). **B**: Onchocerciasis treatment areas (Source: APOC).

Dr Hopkins presented a rationale for partnering with onchocerciasis: the diseases affect overlapping populations, have almost identical disease agents, recommend similar treatment, employ similar M&E methods, and face common challenges. While countries where LF and onchocerciasis are endemic completely overlap, co-endemicity only occurs in some districts (Figure 6).

The LF and the onchocerciasis parasites can be difficult to distinguish, a difference which does not matter for treatment, but does when monitoring progress toward elimination. During preventive chemotherapy, both diseases use ivermectin, while the LF programme adds albendazole. For M&E, both disease programmes need better diagnostics, as skin snips for onchocerciasis and night blood films for LF are not popular and existing serological tests require additional validation. There is a related need to build human and laboratory capacity to carry out entomological and parasitological surveys in both programmes. In addition, existing M&E methods are costly and would need to include parasitological, entomological and serological surveys for onchocerciasis elimination.

In terms of challenges common to both programmes, there are mapping gaps for both LF and onchocerciasis, as well as cross border issues in conflict areas, such as South Sudan and neighbouring countries. Both programmes are lagging behind goals for scaling up. Particularly if onchocerciasis moves towards an elimination strategy, there are 18 million people (not including the Central African Republic, Sudan, and the Democratic Republic of Congo) who live in areas with a nodule prevalence between 5-15% that are not currently being treated. Eighty percent of the people not yet covered by LF treatment live in nine countries, many of which are either treating for onchocerciasis, such as Nigeria, or have found new areas of onchocerciasis endemicity that also need treatment, such as in Ethiopia. Finally, loiasis presents a major obstacle for both programmes in co-endemic areas in central Africa, such as Cameroon, the Democratic Republic of Congo, Congo, Gabon, and Equatorial Guinea, with 30 million people at-risk for loiasis (based on those living in areas with greater than 20% on the RAPLOA scale). In areas where onchocerciasis is meso- or hyper-endemic and co-endemic with loaisis, treatment can still occur. However, given that only 1-2% of people might be infected in areas where LF treatment is prescribed, it is too risky to treat with ivermectin in areas with loiasis, given the chance of serious adverse events due to high levels of *Loa* microfilaremia. The LF programme is now moving forward with a strategy of using twice annual albendazole and bednets in these areas.

Dr Hopkins concluded that there are many opportunities for synergy including integrated mapping, joint training, combined treatment registers, simultaneous MDA, joint M&E, and joint epidemiological evaluations. He challenged the group to not just see the benefit in exploiting synergies for the two programmes, but to see the LF and onchocerciasis activities as one programme in Africa.

#### Extending the benefits

Dr David Addiss (Children Without Worms, USA) presented a summary of ancillary benefits from the LF MDA programme. The LF programme has given the STH community a bold vision of elimination, with a platform for integrated NTD activities related to MDA and M&E. From 2001 to 2011, LF MDA included two billion treatments with albendazole that also treated STH. For example, in 2010, WHO reported that 57% of the school-aged children treated for STH were given these medicines through national LF programmes. In areas with LF programmes, entire communities are treated for LF, which likely gives a greater impact than those areas that only implement school-based STH treatment. In terms of M&E, the LF TAS platform could be used to collect information on STH as well.

However, Dr Addiss cautioned that often the benefits of LF programme have not been fully extended to those with LF-related disease. The rationale for the two pillars of the LF programme stems from the wording of the World Health Assembly resolution – elimination as a public-health problem – which includes those with clinical disease, and not just infection. Providing care for LF-related disease has also been shown to increase acceptability and compliance with MDA. Furthermore, providing care for those with LF-related disease was considered the right and compassionate thing to do. But as of 2010, only 27 (33%) of endemic countries had active morbidity management programmes.

The four basic components of NTD control include preventive chemotherapy (which is usually the largest component and often has a direct clinical benefit); vector control; water, sanitation and hygiene; and clinical care. In the LF programme, morbidity management and disability prevention (MMDP) lags behind PC, while in the STH programme, the water, sanitation and hygiene activities likewise take a back seat to PC. The trachoma programme, however, has a more balanced approach with the four components of the SAFE strategy – surgery, antibiotics, facial cleanliness, and environmental improvement. For the LF programme to truly achieve elimination, it needs to be both internally integrated in terms of MDA, vector control and MMDP activities, as well as externally integrated with other health programmes. A model for how MMDP can be integrated externally has been described in the Legs to Stand On framework, which identifies the common elements of treatment across diseases such as LF, diabetes, leprosy, and Buruli ulcer.

Dr Addiss related another benefit of the GPELF in context of a quote from Bill Foege about ‘seeing the faces’ behind everything we do - i.e., having a compassionate response to suffering. The GPELF represents a compassionate response to LF-related suffering, and has included the three main elements of compassion, which include: i) awareness of suffering, ii) empathy, and iii) action. Publications focusing on the awareness of suffering include studies of psychological distress and disability caused by LF-related diseases. In terms of emotional attunement with patients, he related the power of the personal story - of 'seeing the faces' for those who work in LF elimination. Finally, compassion must lead to action. The magnitude of action in the global LF programme is, by any standard, impressive. Although the GPELF is a great example of a ‘mass uprising of compassion’, the expression of that compassion faces several important challenges. It is difficult to keep seeing the faces when working with populations in the millions and billions, and across great geographical distances. In addition, a global programme requires acting through complex systems of institutions, each with its own agenda, and with competing motivations. Dr Addiss challenged the group to try to maintain focus on interrupting LF transmission while expanding its peripheral vision to include those with LF disease; to unite the LF programme activities across different sectors in the health system; to see faces and numbers at the same time; and to combine compassion for individuals with action at the population level.

#### Two programmes: a shared goal – LF and trachoma

Dr Teshome Gebre (International Trachoma Initiative (ITI), Ethiopia) discussed the shared goals and commonalities between LF and trachoma. Both diseases are acquired in childhood, with terribly painful and severely incapacitating complications that appear later in life. In Africa, there are mapping gaps in nine countries for LF and 19 for trachoma; coordinated mapping could occur in areas of potential overlap to leverage resources. Both programme strategies include MDA and morbidity management, while trachoma also includes the preventive activities of face washing and environmental improvement. In order to achieve trachoma goals by 2020, there is a backlog of 4.6 million trichiasis patients that need surgery, which means scaling up from 160,000 surgeries to 500,000 surgeries a year. While more than 200 million trachoma treatments have been delivered since 1998, 380 million more are needed before 2020. Dr Gebre discussed various ideas for linking the programme interventions including: i) empowering CDDs to handle all MDA through joint planning, training, and implementation; ii) co-administration of drugs where feasible, depending on results of clinical trials underway for ivermectin, albendazole and azithromycin; iii) joint M&E, and iv) initiation of regional, sub-regional, and national NTD coordination mechanisms or annual reviews which are smaller and more technical, using the sub-regional forums to address cross-border issues. Beyond just LF and other NTDs, he proposed links with HIV/AIDS, tuberculosis, and malaria, such as a ‘MalTra’ week approach to reach several million people with MDA and bednets in Ethiopia.

He then discussed the unresolved issues that are hampering achievement of goals, such as gaps in behavior change, hygiene and sanitation, and effective community mobilization strategies. There are concerns with results of MDA coverage surveys; for example, Ethiopia found less than 49% coverage in surveys when health facilities reported over 85% coverage. This has translated into impact assessments that have shown that very few districts showed infection levels of TF less than 10% after three or five rounds and so must continue MDA. Finally, the NTD community needs to find the most effective level of integration and coordination among partners and donors at all levels. He cautioned that if the LF and trachoma programmes do not start doing things differently the 2020 elimination goal will not be achieved.

#### A partnership with malaria programmes

**Figure 7:**
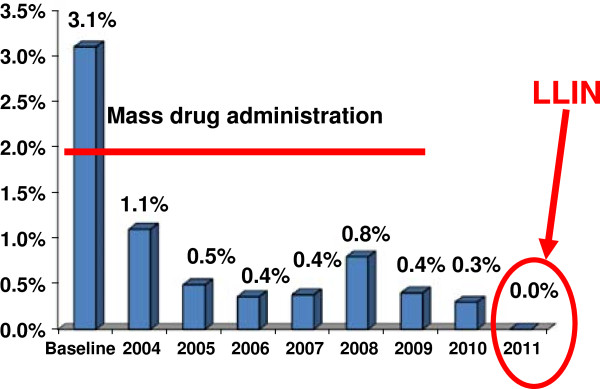
**Average mosquito LF infection rates in nine sentinel villages.** Legend: Based on >46,000 dissections. blue rectangle = Infected.

Dr Richards discussed the Nigerian experience in linking LF with the malaria programme, where *Anopheles gambiae* is the primary vector of both diseases. A 2011 WHO position statement on integrated vector management (IVM) made the case that the policy of using IVM to control malaria and LF makes epidemiologic sense [10]. In Plateau and Nasarawa States in Central Nigeria, the Carter Center has been implementing LF activities integrated with onchocerciasis, schistosomiasis and malaria since 1998. During that period the programme has dissected over 80,000 mosquitoes as part of LF entomological monitoring, using the percentages of any larval stage (L1, L2, and L3 but not including mf stages) and only infective stage larvae (L3) as the critical indicators. The programme saw mosquito rates of infection with any stage larvae decrease during MDA, for an average reduction of 85% from 3.1% to 0.3% in 2010, after at least 7 years of MDA (Figure 7). L3 also were sporadically found in dissections.

In 2010, several million long-lasting insecticidal nets (LLINs) – two nets for every household - were distributed in Plateau and Nasarawa States as part of a massive campaign throughout Nigeria to distribute over 65 million LLIN. A year after LLIN distribution, entomological monitoring in LF sentinel villages recorded 0% mosquitoes infected with L3 larvae, as well as 0% mosquitoes infected with any larval stage. Preliminary results from 2012 are also showing no infected mosquitoes. Thus, there is synergy between LLIN and MDA.

In southeastern Nigeria, where MDA cannot be provided due to concerns about adverse reactions from co-endemic loiasis infection, the Carter Center has studied the entomological impact of LLIN alone (without MDA) in reducing LF transmission. The use of LLIN alone showed an 84% reduction in all larval mosquito infection rates, compared to areas with LLINs and MDA, where 100% reduction was shown. In 2011 in both these areas, no L3 stage larvae were found. This indicates that transmission of LF has stopped – although in LLIN areas it might not be interrupted since halting LLIN use could result in a rapid return of transmission.

Over 90% of Nigeria has been mapped for LF, with over 106 million people found to be at risk in 541 local government areas (LGAs). However, only 19% of LGAs are currently implementing MDA for LF. In contrast, almost all states completed the distribution of LLINs in 2012, and will complete the job in early 2013. If the results from the entomological impact studies hold true elsewhere in areas with good LLIN coverage in Africa, LF programmes can celebrate a major impact on LF transmission now, despite the fact that MDA scale-up is lagging. GAELF partners should encourage, and indeed help, the malaria programme reach its LLIN coverage and usage goals, as it is to the LF programme’s advantage!

Building on advocacy efforts using political figures such as former head of state General Dr Yakuku Gowon, the Carter Center sponsored a national combined LF and malaria programme meeting in March 2012. The meeting presented the entomological monitoring findings, planned scale up of both LF and malaria activities, and created awareness about the benefits of integration, such as the fact that infection with intestinal worms can worsen malaria outcomes. Another benefit is that LF MDA, which intensively targets hookworm through distribution of albendazole to entire communities, results in lower anemia prevalence. Severe anemia accounts for half of all malarial deaths in African children and women, co-infection with malaria and intestinal worms has been shown to result in greater anemia. In addition, LF community-based networks can be used by the malaria programme to mop up LLIN distribution, and implement annual monitoring of LLIN ownership, usage and condition. Finally, behaviour change communication can be strengthened by showing the protection offered by LLINs from both malaria and LF, as focus groups showed that adults are more motivated to sleep under nets to protect themselves from lymphoedema and hydrocele than from malaria fever, which is seen as an infection of young children.

## Discussion

Dr Adiele Onyeze (WHO Africa Regional Office) asked how the global LF community could scale up these approaches at national or regional levels. He related the WHO African Region strategy of the ‘4 1’s approach’ that includes common partnership, planning, delivery and M&E, with a goal of making life better for fellow human beings at risk or already infected. The African Region’s priority is to have NTD national plans and fora by 2013, with new regional programme review group mechanisms at a regional level. The most difficult component of the NTD work in Africa goes beyond the availability of drugs and tools to figuring out how to bring various targets, organizations and personalities together.

Participants agreed that there is a moral imperative to look at synergies in collaboration and coordination. It is important to clearly state the LF community’s needs from other programmes, e.g. what exactly do we want from the malaria, water, morbidity and health system strengthening programmes and organizations? We need to articulate how the LF programme can help other programmes achieve their goals. The LF programme needs strong advocates and quality data to persuade other programmes that “we are not just there for the money”. The challenges of translating the good examples of collaboration in these presentations into other country settings was discussed, including the difficulties of replication in different settings, and the need for new programmes to have access to this information.

### GAELF: the next chapter

Moderator: Dr Mwele Malecela.

Dr Malecela (National Institute for Medical Research, Tanzania) opened the final session by sharing a Swahili saying: ‘in order to see how far you have come, look back and see where you were’. She reflected that GPELF has made great progress since 2000 – with stronger national programmes, great country leadership, a focus on countries and the patients, and an acknowledgement of the importance of good data and research.

#### Strategic partnerships

Dr Neeraj Mistry (Global Network for NTDs, USA) discussed the key elements to strategic partnerships at a country level, including trust, clear objectives, and specific roles and responsibilities. Contributions to a LF programme should focus on partner competencies, such as involving business people who have better training in logistics and distribution than medical doctors. Programmes need to understand the motivation that each partner has to be involved, whether it is the use of their logo, a free t-shirt, or raising their public profile. For programmes (and partnerships) to work, charismatic leadership is necessary to convince governments and partners to support NTD work. The GAELF should cultivate national programme leaders who have the ability and trust to negotiate partnerships at country level.

He then offered some examples of potential partnerships, such as car dealerships lending vehicles during MDA, mobile phone companies donating costs of texting MDA reports, cinema groups showing awareness raising short movies in mobile cinemas in rural areas, local artists or celebrities advocating for NTDs, or companies donating t-shirts. Dr Mistry recommended that national programme reviews include outsiders from business or media in order to get a different perspective on programme success and challenges. He mentioned that a full assessment of the links between HIV/AIDS, tuberculosis, and malaria and NTDs has been sent to the Global Fund for AIDS, Tuberculosis and Malaria (GFATM) to encourage them to approve proposals which include NTD activities. Finally, he implored the group to use its anger at injustice to advocate on behalf of NTDs as we are the only voice of many of these communities.

#### Future of GAELF in post-London declaration environment

Professor David Molyneux (Liverpool School of Tropical Medicine Centre for Neglected Tropical Diseases, UK) discussed the role of GAELF in the greater context of a changing global health, economic and political environment. He highlighted steps in the evolution of the fight against LF, including: pharmaceutical company support; BMGF seed funding; delineation of the role of the GAELF in coordination, resource mobilization and advocacy; emergence of the concept of NTDs; and USAID support.

Since 2000, with support from endemic countries, BMGF, DFID, USAID and others, there have been 2 billion cumulative treatments for LF, one of the most rapidly upscaling public health programmes. GAELF has been used as an umbrella to capture a diversity of partners, learn from country experience, and focus on a particular goal, while recognizing the complexity of problem. GAELF’s strengths are in its loose structure, ability to speak independently, and as a forum for communication between country programmes, researchers and donors. However, more funds are needed to expand morbidity activities, meet elimination goals, and implement critical operational research. The Representative Contact Group is not yet fully representative of the broader constituency. Innovative funding mechanisms, whether centralized funding at the World Bank, from APOC, the GFATM or other models, need to be explored.

Professor Molyneux expressed his vision of keeping GAELF focused on LF and the elimination goal, but adapting to upscale PC for all NTDs given that if LF reaches its target for MDA, a high proportion of PC objectives will be met as well. Instead of one NTD alliance, GAELF would be one in a mix of alliances that would link to the others at different levels, through articulating specific operational research priorities, global advocacy, and capacity building for data management, logistics and clinical issues.

## Discussion

The possibility of having a resident technical advisor in place for key countries (following the example of the guinea worm eradication programme), who sits with the national programme but has the freedom to talk with other ministries, private sector and stakeholders was discussed. Other methods of providing support included i) continuing forums like GAELF to ensure opportunities for discussion among many stakeholders, ii) twinning successful countries with those needing assistance or just starting, and iii) broadening GAELF’s reach to include more African and Indian research centres and universities. While participants agreed with keeping the GAELF as an LF-specific alliance, the idea was put forward of a three-in-one concept of combining parts of LF, STH and onchocerciasis meetings or holding them back-to-back.

### Next steps

Dr Lammie introduced the newly elected Executive Group members: Patrick Lammie, Moses Bockarie, Frank Richards, Adrian Hopkins, Charles Mackenzie, and Njeri Wamae. He then thanked all the speakers for preparing thought-provoking, quality presentations and listed five key recommendations that had emerged from the meeting:

1. Inspirational stories from countries showed clear examples of the quality of country leadership in the GAELF, with leaders who are problem solvers and have the ability to motivate governments and partners to contribute. Since good programme managers make good programmes, support needs to continue to these programme managers. The idea of a mentorship programme, perhaps through the WHO capacity building working group, in which new programme managers could shadow more experienced managers through an MDA cycle, should be explored. In addition, the idea of resident advisors should be taken forward, especially in countries with a single NTD programme manager.

2. The concept of integration has changed from a potential threat to the GPELF to an opportunity to scale up LF, as well as other NTD activities. Countries provided examples from building LF MDA onto onchocerciasis CDD in Sierra Leone and Liberia to child health days in the Philippines to lowering LF prevalence through use of bednets in Nigeria to the use of LF MDA as the single largest mechanism for STH delivery. However, evaluation of the impact of integration is missing, with priorities to strengthen evaluation capacity and operational research in mapping, elimination criteria and post-MDA surveillance needed.

3. The willingness to see the faces in the data is a clear challenge. Thus far, in many countries, the programmes have been highlighting people with disability to leverage community compliance with MDA without providing care for them. This needs to change by the time of the next GAELF meeting.

4. The single greatest determinant of success at country level has been government commitment. The idea of GAELF representatives visiting key countries with WHO and other donors to galvanize support at country level should be taken forward.

5. There is a challenge to be innovative in the way GAELF is organized, both in terms of the mechanism of engaging the Representative Contact Group and in how it coordinates with other disease-specific and NTD meetings. Anyone with suggestions on how best to ensure greater country participation and engagement, as well as more effective use of meeting time, was encouraged to send an email through the GAELF website.

Dr Lammie expressed gratitude to Don Bundy and the World Bank, Julie Jacobson, and the Global Health Strategies team and the CNTD team for organizing the meetings and to the outgoing Executive Group members for putting together the series of panel discussions. Finally, he gave thanks to the 65 countries represented, and the enormous commitment by donors including Merck & Co. Inc. and GSK, as well as the many partners that have supported NTD programmes at country level and supported partners to attend the meeting.

Don Bundy closed the meeting by sharing his impressions of the NTD and GAELF meetings. In the 1980s, LF control was based on vector control, with the only medical input at the clinical and immunological levels. The community is now too focused on preventive chemotherapy, a change facilitated by the drug donations targeting LF. Since incentives are the key to change, he challenged GAELF to continue exploring the reasons for working together and what information needs to be shared.

### Endnote

^a^When South Sudan became independent in 2011, the number of endemic countries rose from 72 to 73.

## Abbreviations

APOC: African Programme for Onchocerciasis Control; BMGF: Bill & Melinda Gates Foundation; CDC: (United States) Centers for Disease Control and Prevention; CDD: Community drug distributor; CNTD: Centre for Neglected Tropical Diseases (Liverpool); DEC: Diethylcarbamazine citrate; DFID: Department for international development, UK; GAELF: Global Alliance to Eliminate Lymphatic Filariasis; GPELF: Global Programme to Eliminate Lymphatic Filariasis; GSK: GlaxoSmithKline; ICT: Immunochromatographic test; IU: Implementation unit; LF: Lymphatic filariasis; LLIN: Long-lasting insecticide-treated net; MDA: Mass drug administration; MMDP: Morbidity management and disability prevention; M&E: Monitoring and evaluation; NTD: Neglected tropical diseases; PC: Preventive chemotherapy; PNG: Papua New Guinea; STH: Soil-transmitted helminthiases; TAS: Transmission assessment survey; USAID: United States Agency for International Development; WHO: World Health Organization.

## Competing interests

The authors declare that they have no competing interests.

## Supplementary Material

Additional file 1 Lists of participants. Click here for file
