# Supplementary material for: Seventh meeting of the Global Alliance to Eliminate Lymphatic Filariasis: reaching the vision by scaling up, scaling down, and reaching out
Source: Parasit Vectors. 2014 Jan 23;7:46. doi: 10.1186/1756-3305-7-46 (PMC3909380; doi:10.1186/1756-3305-7-46)
Supplement: Additional file 1 — Lists of participants. [file 1756-3305-7-46-S1.doc]

# Supplementary material

## List of participants

**Argentina**

Jorge Arguello, Ambassador of the Argentine Republic, Embassy of Argentina, Argentina. Email: [ambassador@embassyofargentina.us](mailto:ambassador@embassyofargentina.us)

**Bangladesh**

Be-Nazir Ahmed, Director General of Health Services, Ministry of Health and Family Welfare, Bangladesh. Email: [dbenazirahmed@yahoo.com](mailto:dbenazirahmed@yahoo.com)

Israt Hafiz, Technical Consultant, Filariasis Elimination Program, Disease Control Unit, Directorate General of Health Services, Dhaka, 1206, Bangladesh. Email: [israthafiz@yahoo.com](mailto:israthafiz@yahoo.com); Tel: +88 01552 415 070; Fax: +88 02986 2994

Rouseli Haq, LF Programme Manager, Ministry of Health and Family Welfare, Dhaka, Bangladesh. Email: [dr.rouselihaq@gmail.com](mailto:dr.rouselihaq@gmail.com)

**Benin**

Etienne Batcho, Coordonnateur du Programme National de Lutte Contre les Maladies Transmissibles, Ministère de la Santé, Benin. Email: [wbatcho@yahoo.fr](mailto:wbatcho@yahoo.fr); Tel: +229 9702 7728

**Brazil**

Regiane Cardoso, SVS / Health Ministry, Brazil. Email: [regiane.paula@saude.gov.br](mailto:regiane.paula@saude.gov.br)

Elaine Ignotti, Doctor, Ministry of Health, Brazil. Email: [eignotti@uol.com.br](mailto:eignotti@uol.com.br)

Rosa Castália Soares, Coordinator of Leprosy and Diseases under Elimination Program, Ministry of Health, Brazil. Email: [rosa.castalia@saude.gov.br](mailto:rosa.castalia@saude.gov.br)

**Burkina Faso**

Francois Marie Didier Zoundi, Ministry of Budget, Burkina Faso. Email: [francois.zoundi@finances.gov.bf](mailto:francois.zoundi@finances.gov.bf)

Windtare Roland Bougma, NPELF Coordinator, Ministry of Health, Burkina Faso. Email: [wrolandbougma@yahoo.fr](mailto:wrolandbougma@yahoo.fr); Tel: +226 702 703 33

Inoussa Ouminga, Directeur général de l'économie et de la planification, Ministère de l'Economie et des Finances, Burkina Faso. Email: [jouiminga@yahoo.fr](mailto:jouiminga@yahoo.fr)

Piga Prosper Tapsoba, Director of Administration and Finance, Ministry of Health, Burkina Faso

Email: [tapspiga@yahoo.fr](mailto:tapspiga@yahoo.fr)

**Burundi**

Sabine Ntakarutimana, Minister, Burundi Ministry of Public Health, Burundi. Email: [ntakarutimana2006@yahoo.fr](mailto:ntakarutimana2006@yahoo.fr)

Onésime Ndayishimiye, Director, Burundi Ministry of Public Health, Burundi. Email: [ndayones@yahoo.fr](mailto:ndayones@yahoo.fr); Tel: +257 799 100 36 or +257 77 735 102

**Cameroon**

Ondobo Andze Gervais, Inspector of Health Services, Ministry of Public Health, Cameroon

Email: [andzegervais@yahoo.fr](mailto:andzegervais@yahoo.fr)

Alphonse Um Boock, NTDs regional coordinator, Fairmed Foundation, Cameroon. Email: [umboock@yahoo.fr](mailto:umboock@yahoo.fr)

**Cote d’Ivoire**

Aboulaye Meite, LF, Schisto and STH Programme Manager, Ministry of Health, Cote d’Ivoire. Email: [aboulaye_meite77@yahoo.fr](mailto:aboulaye_meite77@yahoo.fr); Tel +225 6729 0000

**Democratic Republic of Congo**

Benoit Kebela Ilunga, Directeur de la Direction de la Lutte contre la Maladie, Ministère de la Sante, DRC

Email: [Kebelailunga@gmail.com](mailto:Kebelailunga@gmail.com)

**Dominican Republic**

Manuel Gonzales, National Manager of DR PELF, CENCET, Dominican Republic. Email: [manuelgonpe@gmail.com](mailto:manuelgonpe@gmail.com)

**Ethiopia**

Kesetebirhan Admasu Birhane, Minister for Health, Federal Ministry of Health, Ethiopia. Email: [kesetemoh@gmail.com](mailto:kesetemoh@gmail.com)

Hassane Mahamat, Coordinator, AU_PATTEC, Ethiopia. Email: [hassanehm@africa-union.org](mailto:hassanehm@africa-union.org)

**Egypt**

Reda Ramzy, Professor, National Nutrition Institute, General Organization for Teaching Hospitals &

16 Kasr El Aini St, Cairo 71556, Egypt. Email: [reda_m@masrawy.com](mailto:reda_m@masrawy.com)

**Gabon**

Julienne Atsame, Directeur Maladies Parasitaires , Ministère de la Santé, Gabon. Email: [ntseme2007@yahoo.fr](mailto:ntseme2007@yahoo.fr)

**Ghana**

Irene Ayi, Research Fellow, Memorial Institute for Medical Research, University of Ghana, PO Box LG581, Legon, Accra, Ghana. Email: [IAyi@noguchi.mimcom.org](mailto:IAyi@noguchi.mimcom.org)

Nana-Kwadwo Biritwum, Programme Manager, NTD Control Programme, Ghana Health Service, PO Box MB-190, Accra, Ghana. Email: [nkadbiritwum@gmail.com](mailto:nkadbiritwum@gmail.com) or [nanakwadwo@ghsmail.com](mailto:nanakwadwo@ghsmail.com) ; Tel: +233 20 823 2286; Fax: +233 21 226 739

Daniel A Boakye, Head of Parasitology Department, Noguchi Memorial Institute for Medical Research, University of Ghana, PO Box LG581, Legon, Accra, Ghana. Email: [dboakye@noguchi.mimcom.org](mailto:dboakye@noguchi.mimcom.org); Tel: +233 302 501 178 or +233 302 502 182

Johnny Gyapong, Pro Vice Chancellor, University of Ghana, PO BOX LG571, Legon, Accra, Ghana

Email: [jgyapong@ug.edu.gh](mailto:jgyapong@ug.edu.gh); Tel: +233 302 213 820 ext 2711 or +233 244 265 081

Sunny Doodu Mante, Urologist, African LF Morbidity Project, PO Box KB 282, Korle-Bu, Accra, Ghana

Email: [sunnymante44@yahoo.com](mailto:sunnymante44@yahoo.com); Tel: +233 246 090 078

**Haiti**

Abdel Direny, Senior Program Manager / NTD Advisor, IMA World Health, Haiti. Email: [abdeldireny@imaworldhealth.org](mailto:abdeldireny@imaworldhealth.org); Tel: +509 2942 1513

**India**

Manokaran Gurusamy, Senior Consultant, Apollo Hospital, India. Email: [dr.g.mano@gmail.com](mailto:dr.g.mano@gmail.com)

Jonathan Rout, Project Officer, IMA / CASA, India. Email: [jroutcasa@gmail.com](mailto:jroutcasa@gmail.com)

Kapa Ramaiah, Consultant on NTDs, Control and Neglected Tropical Diseases, India. Email: [ramaiahk@yahoo.com](mailto:ramaiahk@yahoo.com); Tel: +22 791 3862

Pradeep Srivastava, National Vector Borne Disease Control Programme, Ministry of Health and Family Welfare, 22 Sham Nath Marg, New Dehli 100 054, India. Email: [pkmalaria@yahoo.co.in](mailto:pkmalaria@yahoo.co.in); Tel: +11 239 68576

**Indonesia**

Rita Kusriastuti, Director of Vector Borne Disease Control, Ministry of Health Republic of Indonesia, Jln. Percetakan Negara No.29, Jakarta Pusat, Indonesia. Email: [ritakus@yahoo.com](mailto:ritakus@yahoo.com); Tel: +62 214 287 7586; Fax: +62 21 424 7573

**Kenya**

Mary Amuyunzu-Nyamongo, Executive Director, African Institute for Health and Development, Kenya

Email: [Mnyamongo@aihdint.org](mailto:Mnyamongo@aihdint.org)

Doris Wairimu Njomo, Research Scientist, Kenya Medical Research institute, 54840, 00200, Kenya

Email: [dnjomo@kemri.org](mailto:dnjomo@kemri.org); Tel: +254 020 272 2541; Fax: +254 020 272 0030

**Kiribati**

Teiti Bwenawa, Filariasis & Deworming Program Manager, WHO SSA, Kiribati. Email: [bwenawat@wpro.who.int](mailto:bwenawat@wpro.who.int); Tel: +686 28317

**Liberia**

Karsor Kollie, Programme Manager, Ministry of Health, Liberia. Email: [tanue15@yahoo.com](mailto:tanue15@yahoo.com); Tel: +231 886 532 903

Louise Kpoto, Epidemologist, Ministry of Health and Social Welfare, Liberia. Email: [lkpoto@gmail.com](mailto:lkpoto@gmail.com)

Marnijina G Moore, Programme Manager, Ministry of Health and Social Welfare, Liberia. Email: [mgeorgiamoore@gmail.com](mailto:mgeorgiamoore@gmail.com); Tel: +231 886 586 924

Charles Mwansambo, Secretary for Health, Ministry of Health, Malawi. Email: [cmwansambo@gmail.com](mailto:cmwansambo@gmail.com)

**Malawi**

Khumbo Kachali, Vice President / Minister for Health, Malawi. Email: [kachalikhumbo@yahoo.com](mailto:kachalikhumbo@yahoo.com)

Square Zakariya Mkwanda, LF Programme Manager, Ministry of Health, Box 30377, Lilongwe, Central Africa, Malawi. Email: [smkwanda@yahoo.com](mailto:smkwanda@yahoo.com); Tel: +265 8888 54425

**Malaysia**

Rita Kusriastuti, Intensified and Integrated Malaria Control, Malaysia. Email: [mila_mayangsari@yahoo.com](mailto:mila_mayangsari@yahoo.com)

**Mozambique**

Nazira Abdula, Vice Minister of Health, Ministry of Health, Mozambique. Email: [nazvabd@yahoo.com.br](mailto:nazvabd@yahoo.com.br)

Olga Amiel, Head of Department of NTD and LF National Coordinator, Ministry of Health, Mondlane/Salvador Allend Av. Nu. 1008 Maputo, PO Box 264, Mozambique. Email: [olgaamiel@yahoo.com.br](mailto:olgaamiel@yahoo.com.br)

Ricardo Thompson, Senior Researcher, National Institute of Health, Av Eduardo Mondlane, 1008 Maputo 264, P.O Box 264, Maputo, Mozambique. Email: [rthompsonmz@gmail.com](mailto:rthompsonmz@gmail.com); Tel: +258 823 0600 36

**Myanmar**

Ni Ni Aye, Deputy Director (Dengue / Filariasis), Department of Health, Naypyitaw, Myanmar

Email: [niniaye2009@googlemail.com](mailto:niniaye2009@googlemail.com)

**Nepal**

Shanker Shrestha, Deputy Health Minister, Ministry of Health, Nepal. Email: [ppdfamd@mos.com.np](mailto:ppdfamd@mos.com.np)

**Netherlands**

Gabrielle Breugelmans, North North Networking Manager, EDCTP, Netherlands. Email: [breugelmans@edctp.org](mailto:breugelmans@edctp.org)

**Niger**

Adamou Bathiri Salissou, Onchoceriasis and Lymphatic Filariasis Programme Manager, Onchoceriasis and Lymphatic Filariasis Programme, Niger. Email: [sadamouba@yahoo.fr](mailto:sadamouba@yahoo.fr); Tel: +227 207 22879

**Nigeria**

Uche Amazigo, Freelance Consultant, Nigeria. Email: [amazigo4@yahoo.com](mailto:amazigo4@yahoo.com)

Abel Eigege, Director, Plateau / Nasarawa Integrated Programs, The Carter Centre, 1 Jeka Kadima Steet, Jos Plateau State, Nigeria. Email: [eigegea@yahoo.com](mailto:eigegea@yahoo.com); Tel: +908 037 022 967

Chukwu Okoronkwo, NTD Programme Officer, Federal Ministry of Health, Nigeria. Email: [chukoro_christ@yahoo.co.uk](mailto:chukoro_christ@yahoo.co.uk); Tel: +234 803 361 98 94

**Papua New Guinea**

Leo Makita, Principal Advisor, Malaria and Vector Borne Diseases, Department of Health, Papua New Guinea. Email: [leo.makita@gmail.com](mailto:leo.makita@gmail.com); Tel: +675 301 3972

Ross Hutton, Manager – Health Services, Oil Search Health Foundation, Papua New Guinea

Email: [ross.hutton@oilsearch.com](mailto:ross.hutton@oilsearch.com); Tel: +675 322 5597

**Philippines**

Leda Hernandez, Division Chief, Department of Health, Philippines. Email: [dr_ledamher@yahoo.com](mailto:dr_ledamher@yahoo.com); Tel: +900 632 711 6808

**Rwanda**

Irenee Umulisa, Director of NTD Unit, Rwanda Ministry of Health/ RBC / IHDPC / MOPDD, Rwanda

Email: [umulisa5@gmail.com](mailto:umulisa5@gmail.com)

**Senegal**

Serigne Magueye Gueye, Professor, Universite Cheikh Anta DIOP, Senegal. Email: [smgueye@orange.sn](mailto:smgueye@orange.sn); Tel: +221 338 694 061

**South Sudan**

Mounir Lado, Director for Neglected Tropical Disease Control, Ministry of Health, South Sudan

Email: [mounir_lado@yahoo.co.uk](mailto:mounir_lado@yahoo.co.uk); Tel: +211 955 194603

**Sri Lanka**

Udaya Ranasinghe, Director, Anti Filariasis Campaign, Ministry of Health, Sri Lanka. Email: [usbranasinghe@yahoo.com](mailto:usbranasinghe@yahoo.com)

**Switzerland**

Graeme Bilbe, Director of Research and Development, Drugs for Neglected Tropical Diseases, Switzerland. Email: [gbilbe@dndi.org](mailto:gbilbe@dndi.org)

James Cheyne, Independent Consultant, Health Service Logistics, Switzerland. Email: [james@cheyne.net](mailto:james@cheyne.net)

Janis K Lazdins-Helds, Advisor, Mundo Sano, Switzerland. Email: [lazdinsj@gmail.com](mailto:lazdinsj@gmail.com)

Konji Sebati, Senior Director, World Health Intellectual Property Organisation (WIPO), Switzerland

Email: [konji.sebati@wipo.int](mailto:konji.sebati@wipo.int)

**Tanzania**

Hussein Mwinyi, Hon. Minister, Ministry of Health and Social Welfare, Ministry of Health and Social Welfare, Tanzania. Email: [minihealth@yahoo.com](mailto:minihealth@yahoo.com)

Khalfan Mohammed

NTDs Control Programme Manager, Ministry of Health, Zanzibar, Tanzania. Email: [kamsharjyy@yahoo.com](mailto:kamsharjyy@yahoo.com); Tel: +255 777 432 370

Mwelecele Malecela, Director General, National institute for Medical Research, Tanzania

Email: [mmalecela@hotmail.com](mailto:mmalecela@hotmail.com); Tel: +255 222121 400

Upendo Mwingira, National Coordinator, Ministry of Health, P.O Box 9083, Dar-Es-Salaam, Tanzania

Email: [umwingira@yahoo.com](mailto:umwingira@yahoo.com); Tel: +255 713 262 865

**Timor-Leste**

Telma Joana Corte-Real de Oliveira, International Public Health and Health Management, Ministry of Health Timor-Leste, Timor-Leste. Email: [ninatelma@yahoo.com](mailto:ninatelma@yahoo.com)

**Togo**

Ameyo Monique Dorkenoo, LF Coordinator, Ministry of Health, Togo. Email: [monicadork@yahoo.fr](mailto:monicadork@yahoo.fr)

**Uganda**

Gabriel Matwale, Program Manager, Program to Eliminate LF, Vector Control Division; Ministry of Health, P.O Box 1661, Kampala, Uganda. Email: [gkmatwale@yahoo.com](mailto:gkmatwale@yahoo.com); Tel: +256 414 251 927

Edridah Tukahebwa, Assistant Commissioner Health Services (Vector Control), Ministry of Health, Uganda. Email: [edmuheki@gmail.com](mailto:edmuheki@gmail.com); Tel: +44 256 772 443 659

**United States of America**

William Campbell, Senior Finance Executive, JPMorgan Chase, USA. Email: [bill@nsrpartners.com](mailto:bill@nsrpartners.com)

Philip Coyne, Professor Uniformed Services University, USA. Email: [philip.coyne@usuhs.edu](mailto:philip.coyne@usuhs.edu)

B Fenton Hall, Chief, Parasitology & International Programs Branch, DMID/NIAID/NIH/DHHS, USA

Email: [lhall@niaid.nih.gov](mailto:lhall@niaid.nih.gov)

Julie Hliboki, Consultant, 99 Names Peace Project, USA. Email: [juliehliboki@gmail.com](mailto:juliehliboki@gmail.com)

Stewart Marsden, Director, Product Portfolio/Global Health, BD, USA. Email: [Stewart_Marsden@bd.com](mailto:Stewart_Marsden@bd.com)

Chandrakant Revankar, Consultant in Neglected Tropical Diseases, Freelance Consultancy, USA

Email: [revankarcr@gmail.com](mailto:revankarcr@gmail.com)

**Vietnam**

Do Trung Dung, Medical Doctor, NIMPE, Ministry of Health, Vietnam. Email: [dotrungdung.nimpe.vn@gmail.com](mailto:dotrungdung.nimpe.vn@gmail.com)

**Yemen**

Abdul Samid Al-Kubati, National Focal Point for LF, Ministry of Public Health, PO Box 6593, Taiz, Yemen

Email: [a-samidku@hotmail.com](mailto:a-samidku@hotmail.com); Tel: +967 777 92603

**PRIVATE SECTOR**

**Children Without Worms**

Kim Koporc, Director of Program Implementation, Children Without Worms, Task Force for Global Health, USA. Email: [kkoporc@taskforce.org](mailto:kkoporc@taskforce.org)

Kerry Gallo, Senior Program Associate, Children Without Worms, Task Force for Global Health, USA

Email: [kgallo@taskforce.org](mailto:kgallo@taskforce.org); Tel: +1 404 401 16 52

David Addiss, Children Without Worms, The Task Force for Global Health, 325 Swanton Way, Decatur

GA 30030, USA. Email: [dgaddiss@yahoo.com](mailto:dgaddiss@yahoo.com)

**Eisai Inc**

Fabian Gusovsky, Executive Director, CINO Group, Eisai Inc, USA. Email: [fabian_gusovsky@eisai.com](mailto:fabian_gusovsky@eisai.com)

BT Slingsby, Director, Global Access Strategies, Eisai Inc, Japan. Email: [b-slingsby@hhc.eisai.co.jp](mailto:b-slingsby@hhc.eisai.co.jp)

**EMD Serono**

Frederique Santerre, Head of Global Government Affairs on Health Policy, EMD Serono. Email: [Frederique.Santerre@merckgroup.com](mailto:Frederique.Santerre@merckgroup.com)

**GlaxoSmithKline**

Mark Bradley, Director Scientific Support, GlaxoSmithKline, 980 Great West Road, Brentford, Middlesex TW8 9GS, UK. Email: [Mark.h.bradley@gsk.com](mailto:Mark.h.bradley@gsk.com); Tel: +44 20804 75521

Jorge Carrion, Director Government Affairs Latina, GlaxoSmithKline, USA. Email: [jorge.e.carrion@gsk.com](mailto:jorge.e.carrion@gsk.com)

Joy Cole, Director, GlaxoSmithKline, UK. Email: [joy.2.cole@gsk.com](mailto:joy.2.cole@gsk.com)

Tijana Duric, Director, Supply Planning and Finances, GlaxoSmithKline, UK. Email: [tijana.x.duric@gsk.com](mailto:tijana.x.duric@gsk.com)

Minne Iwamoto, Director, Africa Malaria Partnership, GlaxoSmithKline, 200 N. 16th Street PA 19102 USA. Email: [minne.h.iwamoto@gsk.com](mailto:minne.h.iwamoto@gsk.com); Tel: +1 2157517096

Ellen Strahlman, Global Head Neglected Tropical Diseases, GlaxoSmithKline, UK. Email: [ellen.r.strahlman@gsk.com](mailto:ellen.r.strahlman@gsk.com)

Dennis Romerick Tuazon, Project Officer, GlaxoSmithKline, Philippines. Email: [dennis.g.tuazon@gsk.com](mailto:dennis.g.tuazon@gsk.com)

Andrew Wright, Director Disease Programmes, GlaxoSmithKline, 980 Great West Road, Brentford, Middlesex, TW8 9GS Email: [andy.l.wright@gsk.com](mailto:andy.l.wright@gsk.com); Tel: +44 208 047 5515; Fax: +44 208 047 0684

**ITI**

Teshome Gebre, ITI Regional Representative, Africa, Task Force for Global Health, Ethiopia. Email: [tgebre@taskforce.org](mailto:tgebre@taskforce.org); Tel: +251 911 203 524

PJ Hooper, Sr. Associate Director, External Relations, Task Force for Global Health, USA. Email: [phooper@taskforce.org](mailto:phooper@taskforce.org); Tel: +1 4043711460

**Johnson & Johnson**

Patricia Molina, Vice President, Public Affairs, Johnson & Johnson, USA. Email: [pmolino@its.jnj.com](mailto:pmolino@its.jnj.com)

Steven Silber, Head, Flubendazole Development Program, Janssen Research and Development, Johnson & Johnson. Email: [ssilber@its.jnj.com](mailto:ssilber@its.jnj.com)

**Mectizan Donation Program**

Adrian Hopkins, Director, Mectizan Donation Programme, The Task Force for Global Health,

325 Swanton Way, Decatur, Atlanta, Georgia 30030, USA. Email: [ahopkins@taskforce.org](mailto:ahopkins@taskforce.org); Tel: +1 404 371 1460

Joni Lawrence, Associate Director, Mectizan Donation Programme, The Task Force for Global Health,

325 Swanton Way, Decatur, Atlanta, Georgia 30030, USA. Email: [jlawrence@taskforce.org](mailto:jlawrence@taskforce.org); Tel: +1 404 371 1460

Yao Sodahlon

Senior Associate Director, Mectizan Donation Programme, The Task Force for Global Health,

325 Swanton Way, Decatur, Atlanta, Georgia 30030, USA. Email: [ysodahlon@taskforce.org](mailto:ysodahlon@taskforce.org); Tel: +1 404 371 1460

**Merck & Co Inc**

Janet Vessotski, Director, Corporate Responsibility, Merck & Co Inc, One Merck Drive, Mailstop WS2A – 56, Whitehouse Station, NJ 08889 USA. Email: [janet.vessotskie@merck.com](mailto:janet.vessotskie@merck.com); Tel: +1 732 594 2550

**Sanofi Access to Medicines**

Benedict Blayney, Director of Neglected Tropical Disease Programmes, Sanofi Access to Medicines, France. Email: [benedict.blayney@sanofi.com](mailto:benedict.blayney@sanofi.com)

**International Development Agencies and Donors**

**Bill and Melinda Gates Foundation**

Jan Agosti, Senior Programme Officer, Bill & Melinda Gates Foundation, PO Box 23350, Seattle, WA 98102 USA. Email: [jan.agosti@gatesfoundation.org](mailto:jan.agosti@gatesfoundation.org); Tel: +1 206 7093 331

Alexandra Farnum, Program Officer, Malaria, Bill & Melinda Gates Foundation, PO Box 23350, Seattle, WA 98102 USA. Email: [danijela.korac@gatesfoundation.org](mailto:danijela.korac@gatesfoundation.org)

Lance Gordon, Director, Neglected Infectious Diseases, Bill & Melinda Gates Foundation, PO Box 23350, Seattle, WA 98102 USA. Email: [lance.gordon@gatesfoundation.org](mailto:lance.gordon@gatesfoundation.org)

Julie Jacobson, Senior Project Officer, Bill & Melinda Gates Foundation, PO Box 23350, Seattle, WA 98102 USA. Email: [julie.jacobson@gatesfoundtaion.org](mailto:julie.jacobson@gatesfoundtaion.org)

Thomas Kanyok, Senior Program Officer, Bill & Melinda Gates Foundation, PO Box 23350, Seattle, WA 98102 USA. Email: [thomas.kanyok@gatesfoundation.org](mailto:thomas.kanyok@gatesfoundation.org)

Hannah Kettler, Senior Program Manager, Bill & Melinda Gates Foundation, PO Box 23350, Seattle, WA 98102 USA. Email: [hannah.kettler@gatesfoundation.com](mailto:hannah.kettler@gatesfoundation.com)

Caila Nickerson, Program Assistant, Bill & Melinda Gates Foundation, PO Box 23350, Seattle, WA 98102 USA. Email: [caila.nickerson@gatesfoundation.org](mailto:caila.nickerson@gatesfoundation.org)

**END Fund**

Ellen Agler, CEO, The END Fund, 115 Bloomingdale Avenue, Wayne, PA 19087 USA. Email: [eagler@endfund.org](mailto:eagler@endfund.org)

**FHI360**

Nosheen Ahmad, Program Officer, END in Africa, FHI360. Email: [nahmad@fhi360.org](mailto:nahmad@fhi360.org)

Pou Bolivar, Project Director, FHI360, USA. Email: [bpou@fhi360.org](mailto:bpou@fhi360.org)

**Geneva Global Inc**

Warren Lancaster, International Director, Geneva Global Inc, UK. Email: [wlancaster@genevaglobal.com](mailto:wlancaster@genevaglobal.com); Tel: +44 780 976 7986

Anna Rohwer, Associate Program Director, Geneva Global Inc, USA. Email: [arohwer@genevaglobal.com](mailto:arohwer@genevaglobal.com)

Scott Morey, Senior Program Director, Geneva Global Inc, Switzerland. Email: [smorey@genevaglobal.com](mailto:smorey@genevaglobal.com)

**Global Network for Neglected Tropical Diseases**

Michelle Brookes, Policy Director, Global Network for Neglected Tropical Diseases. Email: [michelle.brooks@sabin.org](mailto:michelle.brooks@sabin.org)

Neeraj Mistry, Managing Director of Global Network for NTDs, Sabin Vaccine Institute, Global Network for Neglected Tropical Diseases, USA. Email: [neeraj.mistry@sabin.org](mailto:neeraj.mistry@sabin.org)

Greg Simon, Senior Technical Officer, Sabin Vaccine Institute, Global Network for Neglected Tropical Diseases, USA. Email: [gregory.simon@sabin.org](mailto:gregory.simon@sabin.org)

Wangechi Thuo, Program Officer, Sabin Vaccine Institute, Global Network for Neglected Tropical Diseases, USA. Email: [wangechi.thuo@sabin.org](mailto:wangechi.thuo@sabin.org); Tel: +1 2028425025

Marcia de Souza Lima, Director, Programs and Operations, Sabin Vaccine Institute, Global Network for Neglected Tropical Diseases, USA. Email: [marcia.desouzalima@sabin.org](mailto:marcia.desouzalima@sabin.org)

**Inter-American Developmental Bank**

Agustin Caceres. Communications Specialist, IABD, USA. Email: [agustinc@iadb.org](mailto:agustinc@iadb.org)

**Izumi Foundation**

Gretchen Stoddard, Program Officer, Izumi Foundation, USA. Email: [stoddard@izumi.org](mailto:stoddard@izumi.org)

**RTI International**

Kalpana Bhandari, ENVISION, RTI International, 701 13^th^ St. NW, Suite 750, Washington, DC 20005, USA

Email: [kbhandari@rti.org](mailto:kbhandari@rti.org)

Molly Brady, NTD Technical Advisor, ENVISION, RTI International, 701 13^th^ St. NW, Suite 750, Washington, DC 20005, USA. Email: [mbrady@rti.org](mailto:mbrady@rti.org); Tel: +1 202 728 1967

Katie Crowley, ENVISION, RTI International, 701 13^th^ St. NW, Suite 750, Washington, DC 20005, USA

Email: [kcrowley@rti.org](mailto:kcrowley@rti.org)

Achille Kabore, Senior Technical Advisor, ENVISION, RTI International, 701 13^th^ St. NW, Suite 750, Washington, DC 20005, USA. Email: [akabore@rti.org](mailto:akabore@rti.org); Tel: +1 202 340 8888

Scott McPherson, ENVISION, RTI International, 701 13^th^ St. NW, Suite 750, Washington, DC 20005, USA

Email: [smcpherson@rti.org](mailto:smcpherson@rti.org)

Eric Ottesen, Director, ENVISION Program / LF Support Center, RTI International, Task Force for Global Health, 325 Swanton Way, Decatur, GA 30030, USA. Email: [eottesen@taskforce.org](mailto:eottesen@taskforce.org); Tel: +1 404 687 5604

Lisa Rotondo, Deputy Director Technical, RTI International Envision, USA. Email: [lrotondo@rti.org](mailto:lrotondo@rti.org); Tel: +1 202 974 7890

**The World Bank**

Donald Bundy, Lead Health and Education Specialist , Human Development Network, The World Bank, 1818 H Street, NW, Washington DC 20433, USA. Email: [dbundy@worldbank.org](mailto:dbundy@worldbank.org); Tel: +1 202 473 3636

Patricio Marquez, Lead Health Specialist Eastern and Southern Africa Region, The World Bank, 1818 H Street, NW, Washington DC 20433, USA. Email: [pmarquez@worldbank.org](mailto:pmarquez@worldbank.org)

Ok Pannenborg, Special Advisor, The World Bank, African Region Human Development, 1818 H Street, NW, Washington DC 20433, USA. Email: [opannenborg@worldbank.org](mailto:opannenborg@worldbank.org); Tel: +1 202 473 4415

Abdo Yazbeck, Lead Economist, Health, The World Bank, USA. Email: [ayazbeck@worldbank.org](mailto:ayazbeck@worldbank.org)

**UK Department for International Development**

Delna Ghandi, Senior Health Advisor, DFID, UK. Email: [D-Ghandhi@dfid.gov.uk](mailto:D-Ghandhi@dfid.gov.uk)

Iain Jones, Economic Advisor, DFID, UK. Email: [i-jones@dfid.gov.uk](mailto:i-jones@dfid.gov.uk)

**UNICEF**

Jennifer Fenley-Duffy, US Fund for UNICEF, Director, Integrated Partnerships, USA. Email: [jduffy@unicefusa.org](mailto:jduffy@unicefusa.org)

**USAID**

Ploi Swatdisuk, Program Analyst, USAID, USA. Email: [pswatdisuk@usaid.gov](mailto:pswatdisuk@usaid.gov)

Marci Van Dyke, Technical Advisor, USAID, USA. Email: [mvandyke@usaid.gov](mailto:mvandyke@usaid.gov)

Emily Wainwright, Senior Operations Advisor NTDs, USAID, USA. Email: [ewainwright@usaid.gov](mailto:ewainwright@usaid.gov)

Angela Weaver, Senior Advisor for Neglected Tropical Diseases, USAID, 307-27, Third Floor, Ronald Reagan building, 1300 Pennsylvania Avenue, Washington DC, 20523 USA. Email: [aweaver@usaid.gov](mailto:aweaver@usaid.gov); Tel: +1 202 712 56 03

**International Non-Governmental Organizations (NGOs)**

**American Leprosy Missions**

Mary Jo Geyer, Program Officer / Consultant, American Leprosy Missions, USA. Email: [maryjogeyer@mac.com](mailto:maryjogeyer@mac.com); Tel: +1 412 024 60 02

Linda Faye Lehman, Technical Consultant, American Leprosy Missions, Disease and Disability Prevention, USA. Email: [llehman@leprosy.org](mailto:llehman@leprosy.org)

**Carter Center**

Frank Richards, Director, Malaria, River Blindness, Lymphatic Filariasis & Schistosomiasis Programs, The Carter Center, Atlanta, GA30307, USA. Email: [frich01@emory.edu](mailto:frich01@emory.edu); Tel: +1 770 488 4511

**Christian Blind Mission**

Martin Kollmann, Programme Director NTDs, Christian Blind Mission, Central Africa Regional Office

PO Box 58004-00200 City Square, Ring Road Parklands, Nairobi, Kenya. Email: [mkollmann@cbmi-nbo.org](mailto:mkollmann@cbmi-nbo.org); Tel: +2 542 037 51798 or +2 647 335 24436

Ijeoma Obidegwu, Monitoring and Evaluation Officer, CBM-US, USA. Email: [iobidegwu@cbmus.org](mailto:iobidegwu@cbmus.org)

**Good Neighbours International**

Yunsuk Ko, Managing Director, Good Neighbours International, Tanzania. Email: [atuan.ko@gmail.com](mailto:atuan.ko@gmail.com)

**Handicap International**

Pierre Brantus, NTD medical consultant, Federation Handicap International, 6 Residence Rousseau

67 Chemin des Bergeronnettes, Prevessin-Moens 01280 France. Email: [brantus.pierre@orange.fr](mailto:brantus.pierre@orange.fr)

**HDI Inc**

Stephanie Richard, Program Manager, HDI Inc, USA. Email: [stephanie@hdi-us.org](mailto:stephanie@hdi-us.org)

Anders Seim, Executive Director and Funder, HDI (Health & Development International), USA

Email: [anders@hdi.no](mailto:anders@hdi.no)

**Helen Keller International**

Mary Hodges, Country Director, HKI, Sierra Leone. Email: [mhodges@hki.org](mailto:mhodges@hki.org); Tel: +232 76845 9625

Charles MacArthur, Director of NTD Control, HKI, 1840 Harpswell Neck Road, Harpswell, ME 04079

Email: [cmacarthur@hki.org](mailto:cmacarthur@hki.org); Tel: +1 207 833 73 44

Victoria Quinn, SVP Programs, HKI, USA. Email: [vquinn@hki.org](mailto:vquinn@hki.org)

Mustapha Sonnie, Program Manager, Neglected Tropical Disease, HKI, Sierra Leone. Email: [msonnie@hki.org](mailto:msonnie@hki.org)

Emily Toubali, Program Manager of NTD Control, HKI, USA. Email: [etoubali@hki.org](mailto:etoubali@hki.org)

Yaobi Zhang, Doctor, HKI, USA. Email: [yzhang@hki.org](mailto:yzhang@hki.org); Tel: +1 447 8888 01010

**IMA World Health**

Sarla Chand, Vice President: Programs, IMA World Health, PO Box 429 , 500 Main Street, New Windsor, MD 21776 USA. Email: [sarlachand@imaworldhealth.org](mailto:sarlachand@imaworldhealth.org); Tel: +1 410 635 8720 or +1 443 244 0540

Ann Varghese, Senior Program Manager, IMA World Health, 500 Main Street, New Windsor, MD 21776, USA. Email: [annvarghese@imaworldhealth.org](mailto:annvarghese@imaworldhealth.org); Tel: +1 410 635 8716

**LEPRA**

Venkata Ranganadha Rao Pemmaraju, Chief Executive, LEPRA Society, India. Email: [ranganadh@leprahealthinaction.in](mailto:ranganadh@leprahealthinaction.in)

**Leprosy Mission, Canada**

Peter Derrick, Executive Director, The Leprosy Mission Canada, Canada. Email: [pderrick@leprosy.ca](mailto:pderrick@leprosy.ca)

Grace Folts, Program Officer, The Leprosy Mission Canada, Canada. Email: [gfolts@leprosy.ca](mailto:gfolts@leprosy.ca)

Deborah Mensah Awere, Program Officer, The Leprosy Mission Canada, Canada. Email: [dmensah-awere@leprosy.ca](mailto:dmensah-awere@leprosy.ca)

Dorothy Nyambi, Team Leader, International Programs, The Leprosy Mission Canada, Canada

Email: [dnyambi@leprosy.ca](mailto:dnyambi@leprosy.ca); Tel : +1 416 566 63 34

Anna Wickendon, Leprosy Mission Canada, Canada. Email: [awickendon@leprosy.ca](mailto:awickendon@leprosy.ca)

**Malaria Consortium**

Ruth Ashton, Technical Officer, Malaria Consortium Africa, Uganda. Email: [r.ashton@malariaconsortium.org](mailto:r.ashton@malariaconsortium.org)

**MAP International**

Ravi Jayakaran, Vice President – Global Programs, MAP International, USA. Email: [Globalprogramsupport@map.org](mailto:Globalprogramsupport@map.org)

**Mission to Save the Helpless**

Francisca Olamiju, Executive Director, MITOSATH, 605 Hospital Place (opp. Green Valley Suites, GRA), 93001 Plateau State, Nigeria.Email: [mitosath@hotmail.com](mailto:mitosath@hotmail.com); Tel: +234 734 64792 or +234 803 3311 8085

**PATH**

Tala De Los Santos, Diagnostics Group Leader, PATH, USA. Email: [tdelossantos@path.org](mailto:tdelossantos@path.org)

**Queen Elizabeth Diamond Jubilee Trust**

Astrid Bonfield. Chief Executive, Queen Elizabeth Diamond Jubilee Trust, UK. Email: [astrid.bonfield@qejubileetrust.org](mailto:astrid.bonfield@qejubileetrust.org)

**Sightsavers International**

Agatha Aboe, Global Trachoma Programme Coordinator, Sightsavers, Ghana. Email: [aaboe@sightsavers.org](mailto:aaboe@sightsavers.org); Tel: +233 244 311 588

Simon Bush, Director Neglected Tropical Diseases, African Alliances and Advocacy, Sight Savers International, 21 Nii Nortel Ababio Street, PO Box KIA 18190 Airport Residential Area, Accra, Ghana

Email: [sbush@sightsavers.org](mailto:sbush@sightsavers.org); Tel: +233 2177 4210 or +233 244 322 885

Caroline Harper, CEO, Sightsavers, UK. Email: [charper@sightsavers.org](mailto:charper@sightsavers.org)

Tom Millar, Operations Director, Trachoma Mapping, Sightsavers, UK. Email: [tmillar@sightsavers.org](mailto:tmillar@sightsavers.org)

**United Front Against Riverblindness**

Daniel Shungu, Executive Director, United Front Against Riverblindness, DRC. Email: [dlshungu@riverblindness.org](mailto:dlshungu@riverblindness.org); Tel: +1 609 771 3674 or +1 609 954 3398

**World Vision**

Azadeh Baghaki, Senior Partnerships and Policy Advisor: Health, Nutrition and WASH, World Vision, Australia. Email: [azadeh.baghaki@worldvision.com.au](mailto:azadeh.baghaki@worldvision.com.au)

Dennis Cherian, Deputy Director, Health and HIV, World Vision Inc, USA. Email:  [dcherian@worldvision.org](mailto: dcherian@worldvision.org)

Happy Kumah, Program Manager, World Vision, USA. Email: [hkumah@worldvision.org](mailto:hkumah@worldvision.org)

**Global Regional Programme Review Group Chairs**

**African Regional Office**

Ricardo Thompson, Senior Researcher, National Institute of Health, Av Eduardo Mondlane, 1008 Maputo 264, P.O Box 264, Maputo, Mozambique. Email: [rthompsonmz@gmail.com](mailto:rthompsonmz@gmail.com); Tel: +258 823 060 036

**Americas Regional Office**

Manuel Gonzales, National Manager of DR PELF, CENCET, Dominican Republic. Email: [manuelgonpe@gmail.com](mailto:manuelgonpe@gmail.com)

**Eastern Mediterranean Regional Office**

Reda Ramzy

Professor, National Nutrition Institute, General Organization for Teaching Hospitals &

16 Kasr El Aini St, Cairo 71556, Egypt

Email: [reda_m@masrawy.com](mailto:reda_m@masrawy.com)

**PACELF**

C.P. Ramachandran, Chairperson, WHO-WPRO-RPRG, 1/63, Off Jalan Tunku, Kuala Lumpur 50480

Email: [ramacp@hotmail.com](mailto:ramacp@hotmail.com); Tel : +603 948 610 12531

**South-East Asia Regional Office**

Nirmal Kumar Ganguly, President & Distinguished Biotechnology Research Professor, Jawaharlal Institute of Postgraduate Medical Education and Research, India. Email: [nkganguly@nii.ac.in](mailto:nkganguly@nii.ac.in)

(NB – will not be in attendance)

**Academic and Research Institutes**

**All India Institute of Medical Sciences**

Sarman Singh, Head, Division of Clinical Microbiology, All India Institute of Medical Sciences, New Delhi, India. Email: [sarman_singh@yahoo.com](mailto:sarman_singh@yahoo.com)

**American University, USA**

Larry Sawers, Professor, American University, USA. Email: [lsawers@american.edu](mailto:lsawers@american.edu)

**Bonn University**

Achim Hoerauf, Chair and Head, Department of Medical Microbiology, University of Bonn Medical Center, Germany. Email: [hoerauf@microbiology-bonn.de](mailto:hoerauf@microbiology-bonn.de)

**Bonn University Hospital**

Ute Klarmann-Schulz, Medical Doctor, IMMIP, University Hospital Bonn, Germany. Email: [ute.klarmann@ukb.uni-bonn.de](mailto:ute.klarmann@ukb.uni-bonn.de)

**CDC**

Stephen Cochi, Senior Advisor, Global Immunization Division, CDC, USA. Email: [scochi@cdc.gov](mailto:scochi@cdc.gov)

Christine Dubray, Medical Epidemiologist, CDC, USA. Email: [ffg5@cdc.gov](mailto:ffg5@cdc.gov)

Karla Feeser, EID Fellow, CDC / APHL, USA. Email: [wsc7@cdc.gov](mailto:wsc7@cdc.gov)

LeAnn Fox, Medical Doctor, Centers for Disease Control and Prevention, CDC, Division of Parasitic Diseases, National Center for Infectious Diseases, Atlanta, GA 30341 USA. Email: [lfox@cdc.gov](mailto:lfox@cdc.gov); Tel: +1 770 488 7560

Patrick Lammie, Senior Scientist, CDC, USA, Email: [pjl1@cdc.gov](mailto:pjl1@cdc.gov)

Monica Parise, Branch Chief, CDC, USA.Email: [mparise@cdc.gov](mailto:mparise@cdc.gov)

Larry Slutsker, Director, Division of Parasitic Diseases and Malaria, CDC, USA. Email: [lms5@cdc.gov](mailto:lms5@cdc.gov)

Kimberly Won, Health Scientist, CDC, USA. Email: [kfw7@cdc.gov](mailto:kfw7@cdc.gov)

**Columbia University**

Martin Chalfie, Professor, Columbia University, USA. Email: [mc21@columbia.edu](mailto:mc21@columbia.edu)

**Erasmus MC, University Medical Center Rotterdam**

Wilma Stolk, Epidemologist, Erasmus MC, University Medical Center Rotterdam, Netherlands

Email: [w.stolk@erasmusmc.nl](mailto:w.stolk@erasmusmc.nl)

**Georgetown University**

Margaret Baker, Assistant Professor, Department of International Health, School of Nursing and Health Sciences, 3700 Reservoir Road NW, Washington DC 20057-1107, USA. Email: [mcb93@georgetown.edu](mailto:mcb93@georgetown.edu); Tel: +1 202 687 4497

Bernhard Liese, Chair, International Health, Georgetown University, 3700 Reservoir Road, Washington DC, DC 20057, USA. Email: [Bhl6@georgetown.edu](mailto:Bhl6@georgetown.edu); Tel: +1 202 687 3254

**Gettysburg College, USA**

Eileen Stillwaggon, Professor of Economics, Gettysbury College, USA. Email: [stillwaggon@gettysburg.edu](mailto:stillwaggon@gettysburg.edu)

**Jazan University**

Maged El-Setouhy, Scientific Director of SARC. Jazan University, Saudi Arabia. Email: [maged.elsetouhy@gmail.com](mailto:maged.elsetouhy@gmail.com); Tel: +966 56 806 9515

**James Cook University**Patricia Graves, Assoc Prof/Director WHO LF/STH/NTD collaborating centre, James Cook University, Australia. Email: [patricia.graves@jcu.edu.au](mailto:patricia.graves@jcu.edu.au); Tel: +1 404 420 3897

**Imperial College London**

Roy Anderson, Professor of Infectious Disease Epidemiology, Imperial College, College Headquarters, London, SW7 2AZ, UK. Email: [roy.anderson@imperial.ac.uk](mailto:roy.anderson@imperial.ac.uk); Tel: +44 20 72176588

Lynsey Blair, Senior Project Manager, Schistosomiasis Control Initiative, Division of Epidemiology, Public Health and Primary Care, Imperial College, London, UK. Email: [l.blair@imperial.ac.uk](mailto:l.blair@imperial.ac.uk)

Lesley Drake, Executive Director, Partnership for Child Development, Imperial College London, UK

Email: [lesley.drake@imperial.ac.uk](mailto:lesley.drake@imperial.ac.uk)

Jane Lillywhite, Managing Director, Partnership for Child Development, Imperial College London, UK

Email: [j.lillywhite@imperial.ac.uk](mailto:j.lillywhite@imperial.ac.uk)

Brie McMahon, Partnerships Manager, Partnership for Child Development, Imperial College London, UK. Email: [b.mcmahon@imperial.ac.uk](mailto:b.mcmahon@imperial.ac.uk)

Kabatereine Narcis, African Capacity Building Advisor, Schistosomiasis Control Initiative (SCI) Imperial College, UK. Email: [vcdmoh@gmail.com](mailto:vcdmoh@gmail.com)

Alexis Palfreyman, Programme Manager, Partnership for Child Development, Imperial College London, UK. Email: [a.palfreyman@imperial.ac.uk](mailto:a.palfreyman@imperial.ac.uk)

Anna Phillips, Programme Manager, Schistosomiasis Control Initiative, Imperial College London, UK

Email: [a.phillips05@ic.ac.uk](mailto:a.phillips05@ic.ac.uk)

**Infectious Disease Research Institute (IDRI), USA**

Steven Reed, President, Founder, and Chief Scientific Officer, Infectious Disease Research Institute (IDRI), USA. Email: [steven.reed@idri.org](mailto:steven.reed@idri.org)

**Kenya Medical Research Institute**

Pauline Mwinzi, Principal Scientist, Kenya Medical Research Institute, Kenya. Email: [pmwinzi@kemricdc.org](mailto:pmwinzi@kemricdc.org)

Doris Njomo, Research Social Scientist, Kenya Medical Research Institute, P.O.Box 54840-00200, KEMRI, Kenya. Email: [dnjomo@kemri.org](mailto:dnjomo@kemri.org); Tel: +202 722 541 Ext 3420

Maurice Odiere, Senior Research Scientist, Kenya Medical Research Institute, Nairobi, Kenya

Email: [modiere@kemricdc.org](mailto:modiere@kemricdc.org)

Njeri Wamae, Chief Research Officer, Centre for Microbiology Research, Kenya Medical Research Institute, Nairobi, Kenya. Email: [nwamae@kemri.org](mailto:nwamae@kemri.org); Tel: +254 202 730409

**Lindsley F. Kimball Research Institute**

Sara Lustigman, Head, Laboratory of Molecular Parasitology, Lindsley F. Kimball Research Institute, USA. Email: [slustigman@nybloodcenter.org](mailto:slustigman@nybloodcenter.org)

**Liverpool School of Tropical Medicine**

Moses Bockarie, Director of CNTD, Liverpool School of Tropical Medicine, Centre for Neglected Tropical Diseases, Pembroke Place, Liverpool, L3 5QA, UK. Email: [mjb12@liverpool.ac.uk](mailto:mjb12@liverpool.ac.uk); Tel: +44 151 705 3343

Alison Blacklock, Programme Administrator, Liverpool School of Tropical Medicine, Centre for Neglected Tropical Diseases, Pembroke Place, Liverpool, L3 5QA, UK. Email: [sara.holmes@liv.ac.uk](mailto:sara.holmes@liv.ac.uk); Tel: +44 151 705 2592

Rinki Deb, Research Assistant, Liverpool School of Tropical Medicine, Centre for Neglected Tropical Diseases, Pembroke Place, Liverpool, L3 5QA, UK. Email: [rinkideb@liverpool.ac.uk](mailto:rinkideb@liverpool.ac.uk); Tel: +44 151 705 3131

Joan Fahy, Programme Manager, Liverpool School of Tropical Medicine, Centre for Neglected Tropical Diseases, Pembroke Place, Liverpool, L3 5QA, UK. Email: [fahy@liv.ac.uk](mailto:fahy@liv.ac.uk); Tel: +44 151 705 3145

Benjamin Koudou, Programme Manager, Liverpool School of Tropical Medicine, Centre for Neglected Tropical Diseases, Pembroke Place, Liverpool, L3 5QA, UK. Email: [gkoudou@liv.ac.uk](mailto:gkoudou@liv.ac.uk); Tel: +44 151 705 2594

Louise Kelly-Hope, Programme Manager, Liverpool School of Tropical Medicine, Centre for Neglected Tropical Diseases, Pembroke Place, Liverpool, L3 5QA, UK. Email: [lkhope@liv.ac.uk](mailto:lkhope@liv.ac.uk); Tel: +44 151 705 3336

David Molyneux, Senior Professorial Fellow, Liverpool School of Tropical Medicine, Centre for Neglected Tropical Disease, Pembroke Place, Liverpool, L3 5QA, UK. Email: [david.molyneux@liv.ac.uk](mailto:david.molyneux@liv.ac.uk); Tel: +44 151 705 3291

Steven Perry, Vice President, LATH / USA, 3527 6th Street (South), Arlington, VA 22204, USA

Email: [Sperry@lath.com](mailto:Sperry@lath.com); Tel: +1 571 312 4728

Maria Rebollo, Programme Manager, Liverpool School of Tropical Medicine, Centre for Neglected Tropical Disease, Pembroke Place, Liverpool, L3 5QA, UK. Email: [mrebollo@liverpool.ac.uk](mailto:mrebollo@liverpool.ac.uk); Tel: +44 151 705 3335

Michelle Stanton, Post Doctoral research Assistant, Liverpool School of Tropical Medicine, Centre for Neglected Tropical Disease, Pembroke Place, Liverpool, L3 5QA, UK. Email: [mstanton@liv.ac.uk](mailto:mstanton@liv.ac.uk); Tel: +44 151 705 2593

Mark Taylor, Professor of Parasitology, Director of AWOL, Liverpool School of Tropical Medicine, Centre for Neglected Tropical Disease, Pembroke Place, Liverpool, L3 5QA, UK. Email: [mjtaylor@liverpool.ac.uk](mailto:mjtaylor@liverpool.ac.uk), Tel: +44 151 705 3112

Brent Thomas, Research Scientist, Liverpool School of Tropical Medicine, Centre for Neglected Tropical Disease, Pembroke Place, Liverpool, L3 5QA, UK. Email: [bthomas@liv.ac.uk](mailto:bthomas@liv.ac.uk), Tel: +44 151 705 3180

**London School of Hygiene and Tropical Medicine**

Anthony Soloman, Senior Lecturer, London School of Hygiene & Tropical Medicine, UK. Email: [anthony.solomon@lshtm.ac.uk](mailto:anthony.solomon@lshtm.ac.uk)

**Lymphatic Filariasis Support Center, Atlanta**

Brian Chu, Research Project Manager, Task Force for Global Health, 325 Swanton Way, Decatur, Atlanta, Georgia 30030, USA. Email: [bchu@taskforce.org](mailto:bchu@taskforce.org); Tel: +1 404 592 1427

Rebecca Mann, Information Analyst, LF Support Center, USA. Email: [rmann@taskforce.org](mailto:rmann@taskforce.org)

Dominique Kyelem, Program Director, Task Force for Global Health, Lymphatic Filariasis Support Center, 325 Swanton Way, Decatur, Georgia 30030 USA. Email: [Dkyelem@taskforce.org](mailto:Dkyelem@taskforce.org); Tel: +1 404 687 5621

Alex Pavluck, Senior Manager of Information Technology, Task Force for Global Health, 325 Swanton Way, Decatur, Atlanta, Georgia 30030, USA. Email: [apavluck@taskforce.org](mailto:apavluck@taskforce.org); Tel: +1 4045 921421

Vasanthapuram Kumaraswami, Associate Director, International Programs, Task Force for Global Health, USA. Email: [Kumaraswami@gmail.com](mailto:Kumaraswami@gmail.com)

**McGill University, Canada**

Roger Pritchard, James McGill Professor, McGill University, Canada. Email: [roger.prichard@mcgill.ca](mailto:roger.prichard@mcgill.ca)

**Michigan State University**

Charles Mackenzie, Professor, Michigan State University, USA. Email: [mackenz8@msu.edu](mailto:mackenz8@msu.edu)

**National Institute of Health, Mozambique**

Ricardo Thompson, Senior Researcher, National Institute of Health, Av Eduardo Mondlane, 1008 Maputo 264 P.O Box 264, Maputo, Mozambique. Email: [rthompsonmz@gmail.com](mailto:rthompsonmz@gmail.com); Tel: +258 823 0600 36

**National Institute for Research in Public Health, Mali**

Mamadou Traoré, Director, National Institute for Research in Public Health, Mali. Email: [traorem@afribonemali.net](mailto:traorem@afribonemali.net)

**National Nutrition Institute, Egypt**

Reda Ramzy, Professor, National Nutrition Institute, General Organization for Teaching Hospitals &

16 Kasr El Aini St, Cairo 71556, Egypt. Email: [reda_m@masrawy.com](mailto:reda_m@masrawy.com)

**Notre Dame University, USA**

James Reimer, Salt Project Director, Haiti Program, University of Notre Dame, 351 Galvin Life Science IN 46628, USA. Email: [Reimerjn@gmail.com](mailto:Reimerjn@gmail.com)

Thomas Streit, Associate Professor, University of Notre Dame, 351 Galvin Life Science IN 46628, USA

Email: [tstreit@nd.edu](mailto:tstreit@nd.edu); Tel: +1 150 987 0746

**Quest International University Perak, Malaysia**

Subhada Prasad Pani, Research Professor & Head of Microbiology & Deputy Dean (Pre-clinical), Faculty of Medicine, Quest International University Perak, Malaysia. Email: [pani.sp@gmail.com](mailto:pani.sp@gmail.com)

**Research!America**

Jennifer Chow, Director, Global Health, Research!America, USA. Email: [jchow@researchamerica.org](mailto:jchow@researchamerica.org)

Eleanor De Honey, Vice President of Policy and Programs, Research!America, USA. Email: [Edehoney@researchamerica.org](mailto:Edehoney@researchamerica.org)

**Swiss Tropical and Public Health Institute**

Randee Kastner, PhD Candidate, Swiss Tropical and Public Health Institute, Switzerland. Email: [randee.kastner@unibas.ch](mailto:randee.kastner@unibas.ch)

Peter Steinmann, Research Associate, Swiss Tropical and Public Health Institute, Switzerland

Email: [peter.steinmann@unibas.ch](mailto:peter.steinmann@unibas.ch)

Chris Stone, Scientific collaborator, Swiss Tropical and Public Health Institute, Switzerland

Email: [c.stone@unibas.ch](mailto:c.stone@unibas.ch)

**Task Force for Global Health**

Mark Rosenberg, President and CEO, The Task Force for Global Health, 325 Swanton Way, Decatur, Atlanta, Georgia 30030, USA. Email: [mrosenberg@taskforce.org](mailto:mrosenberg@taskforce.org); Tel: +1 404 373 4440

**University of Edinburgh**

David Taylor, Professor of Tropical Health, University of Edinburgh, United Kingdom. Email: [David.W.Taylor@ed.ac.uk](mailto:David.W.Taylor@ed.ac.uk)

**University of Sydney**

Peter McMinn, Bosch Professor of Infectious Diseases, Infectious Diseases & Immunology, Sydney Medical School University of Sydney, NSW 2006, Australia. Email: [peter.mcminn@sydney.edu.au](mailto:peter.mcminn@sydney.edu.au); Tel: +61 2 9351 2900 or +61 2 9351 5319

**Washington University**

Alison Krentel, Consultant, Washington University - DOLF project, Washington DC, USA. Email: [amkrentel@yahoo.co.uk](mailto:amkrentel@yahoo.co.uk)

Andrew Majewski, Scientific Project Manager, Washington University School of Medicine, Washington DC, USA. Email: [amajewsk@dom.wustl.edu](mailto:amajewsk@dom.wustl.edu)

Ramakrishna Rao, Associate Professor of Medical Research, Washington University School of Medicine, Washington DC, USA. Email: [rrao@dom.wustl.edu](mailto:rrao@dom.wustl.edu)

Gary Weil, Professor, Division of Infectious Diseases, Washington University School of Medicine, 660 S. Euclid Avenue, Campus Box 8051, St Louis, MO63110-1093, Washington DC, USA

Email: [gweil@dom.wustl.edu](mailto:gweil@dom.wustl.edu); Tel: +1 314 747 5198

**WORLD HEALTH ORGANIZATION (WHO)**

**WHO Headquarters**

Dirk Engels, Coordinator, HTM/NTD/PCT, Department of Control of Neglected Tropical Diseases

20 Avenue Appia, CH 1211 Geneva 27, Switzerland. Email: [engelsd@who.int](mailto:engelsd@who.int); Tel: +41 22 791 3824 or +41 22 791 4777

Kazuyo Ichimori, Scientist, WHO, 20 Avenue Appia, CH 1211 Geneva 27, Switzerland. Email: [ichimorik@who.int](mailto:ichimorik@who.int), Tel: +41 22 791 2767

Lorenzo Savioli, Director, Department of Control of Neglected Tropical Diseases, WHO, 20 Avenue Appia, CH 1211 Geneva 27, Switzerland. Email: [saviolil@who.int](mailto:saviolil@who.int), Tel: +41 22 791 2664

Tony Oka Ukety, Technical officer, WHO, 20 Avenue Appia, CH 1211 Geneva 27, Switzerland

Email: [uketyt@who.int](mailto:uketyt@who.int)

**WHO AFRO**

Amadou Garba, Medical officer/PCT NTD Focal Person, IST/WA, WHO AFRO, Burkina Faso

Email: [garbaa@bf.afro.who.int](mailto:garbaa@bf.afro.who.int)

Adiele Onyeze, Regional Advisor, WHO AFRO, PO Box 06, Dioue, Brazzaville, Democratic Republic of Congo. Email: [onyezea@afro.who.int](mailto:onyezea@afro.who.int); Tel: +242 0534 35272

Louis-Albert Tchuem Tchuente, PCT NY Focal Person, WHO AFRO Intercountry Support Team / ESA, Zimbabwe. Email: [tchuemtchuentel@zw.afro.who.int](mailto:tchuemtchuentel@zw.afro.who.int); Tel: +237 777 07436

Michelle Thulkanam, Partnership, Advocacy & Resource Mobilization Officer, WHO AFRO, Republic of Congo. Email: [thulkanamm@afro.who.int](mailto:thulkanamm@afro.who.int)

**WHO APOC**

Paul-Samson Lusamba-Dikassa, Director, African Onchocerciasis Control Programme (APOC), Burkina Faso. Email: [lusambap@oncho.afro.who.int](mailto:lusambap@oncho.afro.who.int)

**WHO EMRO**

Riadh Ben-Ismail, Regional Advisor, Control of Tropical Diseases, WHO EMRO, Egypt. Email: [ismailr@emro.who.int](mailto:ismailr@emro.who.int)

**WHO PAHO**

Yoaska Acevedo, Intern, Pan American Health Organization, USA. Email: [yaoska.acevedo@gmail.com](mailto:yaoska.acevedo@gmail.com)

Steven Ault, Senior Advisor Neglected Diseases, PAHO WHO, 525 23rd Street NW, Washington DC 20037. Email: [aultstev@paho.org](mailto:aultstev@paho.org); Tel: +1 202 974 3896

Luis Gerardo Castellanos, Coordinator and Senior Advisor, PAHO WHO, USA. Email: [castellanosl@paho.org](mailto:castellanosl@paho.org)

Laura Catala Pascual, Project Support Specialist, PAHO WHO, USA. Email: [catalal@paho.org](mailto:catalal@paho.org)

Marcos Espinal, Area Manager HSD, WHO PAHO, USA. Email: [espinalm@paho.org](mailto:espinalm@paho.org)

Martha Saboya, Specialist NTD, WHO PAHO, USA. Email: [saboyama2@paho.org](mailto:saboyama2@paho.org)

Gabriel Schmunis, Consultant, WHO PAHO, USA.Email: [gabriel.schmunis@gmail.com](mailto:gabriel.schmunis@gmail.com)

**WHO SEARO**

Aditya Prasad Dash, Regional Advisor (Vector Borne & Neglected Tropical Diseases), South East Asia Regional Office of World Health Organisation, India. Email: [dasha@searo.who.int](mailto:dasha@searo.who.int)

**WHO WPRO**

Sung Hye Kim, Scientist, WHO/WPRO, Fiji. Email: [kimsu@wpro.who.int](mailto:kimsu@wpro.who.int)

**Rapporteur**

Molly Brady

NTD Technical Advisor, ENVISION, RTI International, 701 13^th^ St. NW, Suite 750, Washington, DC 20005

Email: [mbrady@rti.org](mailto:mbrady@rti.org); Tel: +1 202 728 1967
